# Engagement Trends in Online Vaccine Content: Longitudinal YouTube Study

**DOI:** 10.2196/88519

**Published:** 2026-06-19

**Authors:** Michele Tizzani, Yelena Mejova

**Affiliations:** 1 Section for Cognitive Systems, Department of Applied Mathematics and Computer Science, Technical University of Denmark, Richard Petersens Plads, Kgs. Lyngby, Capital Region, 2800, Denmark, 45 45 25 30 31; 2 Institute for Scientific Interchange, Turin, Piedmont, Italy

**Keywords:** vaccine hesitancy, YouTube, engagement dynamics, longitudinal analysis, Bayesian modeling, echo chambers, misinformation, social media, temporal patterns, public health policy

## Abstract

**Background:**

YouTube is the primary global video platform, hosting both authoritative health information and vaccine-skeptic viewpoints. However, engagement dynamics remain poorly understood.

**Objective:**

The aim of this study was to investigate the temporal and textual dynamics of engagement of the YouTube viewership with vaccination content, and specifically content that is in favor of or against vaccination. We contextualized these dynamics in the authority signals of the posting channel and the moderation actions taken by the platform.

**Methods:**

We conducted a 6-month daily longitudinal analysis of 7213 vaccine-related YouTube videos (November 2024 to May 2025) mentioning vaccination. We used zero-shot large language model classification with manual verification to classify the video stance toward vaccination, and the stance of their comments toward the video. The engagement and disagreement dynamics were modeled using Bayesian regression.

**Results:**

Our findings show engagement asymmetry between content supporting and questioning vaccination. Vaccine-hesitant videos in our sample receive substantially higher raw engagement (median likes: 40 [IQR 3-846] to 59 [IQR 3-1319]; median comments: 10 [IQR 0-160] to 18 [IQR 0-311] per video versus 3 [IQR 1-15] and 0 [IQR 0-4], respectively, for strongly provaccine content) and moderate normalized engagement rates (true median combined rate: 0.073 [IQR 0.028-0.121] to 0.069 [IQR 0.027-0.118] interactions per view versus 0.026 [IQR 0.007-0.060] for strongly pro-vaccine videos, a 2.5-2.6× difference). Descriptively, vaccine-hesitant videos reach 90% of cumulative views faster (18 [IQR 8-38] days vs 32 [IQR 18-64] days; 44% faster), while negative binomial models that adjust for total engagement volume indicate that approximately 20% of this advantage reflects genuine temporal compression independent of engagement volume. Comment analysis indicated that the vaccine-hesitant videos in our sample foster echo chambers, while the provaccine content attracts battlegrounds. Considering the sources of vaccine-related content, provaccine content tends to originate from organizations, particularly news and health institutions, while vaccine-hesitant discourse is more likely to come from individual creators, even those self-identifying as medical doctors. Moderation, on the rare occasion when it occurs (about 2% of the videos were taken down), comes after engagement saturation, limiting its effectiveness.

**Conclusions:**

Our analysis suggests that the vaccine-hesitant content can dominate YouTube’s engagement ecosystem through rapid early-stage amplification, which has direct implications for public health intervention timing and platform governance policy.

## Introduction

Despite saving millions of lives [1], vaccination has been a contentious public health issue, with hesitancy increasing globally since the COVID-19 outbreak [2,3]. In 2025, the debate around vaccination became embedded in government policy and electoral politics (especially in the United States) following the strong politicization of the topic during the pandemic [4]. While the acute phase of the pandemic has passed and the vaccination debate has been settled in the scientific community, the battle for public opinion is still underway, and it could reshape the long-standing public health policies. The discourse on vaccination is actively shaping the government’s decisions, and in the United States, political movements are gaining traction, promising to “Make America Healthy Again” and framing vaccination as a political stance. Already, the United States is re-evaluating its role as a supporter of vaccine-based medicine both worldwide and domestically. The consequences are in evidence: the largest measles outbreak—since its elimination status was achieved in 2000—has reached 1431 cases and 3 deaths by September 2025 [5].

This shifting policy landscape underscores the urgency of understanding online vaccine discourse. Social media platforms have become central battlegrounds in shaping public opinion, serving as both powerful tools for health communication and vectors for misinformation that can undermine public trust in vaccines [6,7]. Among these platforms, YouTube is a primary source of information for a global audience, making the quality and reception of its health content a matter of critical public concern. In 2025, the Pew Research Center found that the vast majority of US adults (84%) say they have ever used YouTube, and about half of them go on YouTube daily, and that it is the most widely used online platform among US teens [8,9].

Prior research has established that YouTube hosts a mix of credible and misleading vaccine-related content, but none do so longitudinally. Earlier, Basch et al [10] examined the 87 most widely viewed videos around vaccination safety and their creators, finding that 65.5% discouraged the use of vaccines. Then, in 2021, Tang et al [11] created recommendation networks of pro- and antivaccine videos and found that when the user begins their session by searching provaccine keywords, they are recommended more videos by governmental agencies, hospitals, and academic institutions than when users search for antivaccine keywords. More recently, Ng et al [12] “gamified” this analysis by asking real-world participants to intentionally find an antivaccine video when starting from the World Health Organization’s posts, finding that less than 6% of the videos recommended by the algorithm were antivaccine. A most recent dataset—albeit focusing on COVID-19—was created by Jung et al [13] using sock-puppets. They found that 31.5% of the top 10 search results contained COVID-19 misinformation, including that about vaccination. As exemplified above, not only do the studies in the existing literature use heterogeneous research designs, (intentionally) biased queries, and crawling starting points, they are, above all, limited by their *cross-sectional* nature (also including manually annotated surveys such as [14]). They suggest that antivaccination content indeed exists on YouTube but fail to provide the full scope of posted vaccine-related content, and to capture the temporal dynamics of the actual users’ interaction with it.

Nonetheless, within this methodologically disparate landscape, we find a troubling trend: misleading or vaccine-hesitant videos often attract significantly more engagement—in views, likes, and comments—than accurate, provaccine videos [10,11]. This “engagement asymmetry” aligns with broader findings that false news spreads faster and further than true news in online environments [15]. Although YouTube has a medical misinformation policy [16] that advises users not to post information that “contradicts health authority guidance” on the prevention and treatment of diseases, the stricter COVID-19 era policies have been phased out in 2023 [17]. This leaves critical policy-relevant questions unanswered: How quickly does vaccine-hesitant content capture audience attention compared to provaccine content? And what is the nature of the discourse these different types of content foster?

Controversy around vaccination has been shown to be reflected on social media, such as Facebook and Twitter [4,18,19]. Further, Gruzd et al [20] documented Facebook-to-YouTube pathways that contributed to the spread of antivaccination content and formed clusters of densely connected videos. These areas of discourse, wherein information favors a certain stance while other views are excluded, are indicative of echo chambers [21]. Note that the stance may be in favor or in opposition to some topic, such as vaccination, making possible echo chambers containing the majority opinion that disagrees with the medically established vaccination advice. Unfortunately, Gruzd et al [20] did not capture possible disagreements within the comment-level discourse. On the other hand, Ekram et al [22] found that comments on YouTube videos around the human papillomavirus vaccine often mentioned its potential side effects and conspiracy theories. Such spaces, wherein different or opposing points of view are discussed actively, can be viewed as a battleground or a controversy [23]. Unfortunately, Ekram et al [22] did not track the engagement temporally. In this work, we aim to combine quantitative, temporal, and qualitative text engagement signals to discover whether echo chambers or battlegrounds better characterize the vaccination debate on YouTube.

This dynamism is further complicated by the variety of actors posting health-related information on social media. Although public health institutions and researchers have joined the conversation, online influencers hold dominance by amassing vastly larger followings [24]. Such competition for attention is especially acute during emergencies, exemplified by the beginning of the COVID-19 pandemic, when political and commercial interests competed with the public health messaging [25]. The connection between the use of social media and higher vaccine hesitancy during this period has shown a robust negative impact on the vaccination efforts [26,27], which cost an estimated thousands of lives [28,29]. Given the high stakes, it is important to further understand the engagement dynamics around organizational accounts, in comparison to those of individuals who possibly have no medical credentials.

This study contributes a *unique systematic daily longitudinal analysis* of vaccine-related YouTube videos over a 6-month period. By tracking thousands of videos from the day of their upload through their engagement maturation, we provide a fine-grained temporal account of the online vaccine discourse. Unlike previous studies, which may confound engagement patterns with post hoc algorithmic amplification or viral surprises, this study focuses on the earliest phase of a video’s lifecycle—the initial engagement that occurs within the first few days and weeks of upload, when creators’ original reach and audience composition have the most direct influence. Our multifaceted approach combines scalable, large language model (LLM)–based classification of video stances and comment agreement with Bayesian statistical modeling to answer three key questions:

1. *Temporal dynamics*: How do the engagement trajectories of pro- and vaccine-hesitant videos differ over time?
2. *Discourse characterization*: Do these different types of videos cultivate confirmatory “echo chambers” or contentious “battlegrounds” in their comment sections?
3. *Source effects*: How do channel characteristics, such as topic and authority, moderate these discourse patterns?

We provide quantitative evidence that vaccine-hesitant content not only receives 10-fold higher raw engagement volume (and 3.1-fold higher per-view engagement rates) but also reaches its engagement peak faster than provaccine content (44% descriptively; 20% after controlling for engagement volume). Furthermore, through comment-level analysis, we demonstrate that this engagement follows distinct patterns: vaccine-hesitant videos foster agreeable echo chambers, while provaccine videos become contentious battlegrounds characterized by high levels of disagreement. These findings have significant implications for public health policy, platform governance, and science communication, highlighting the narrow window for effective intervention and the urgent need for context-aware communication strategies in an era of heightened vaccine policy contention.

## Methods

### Data Collection

**Figure 1.**
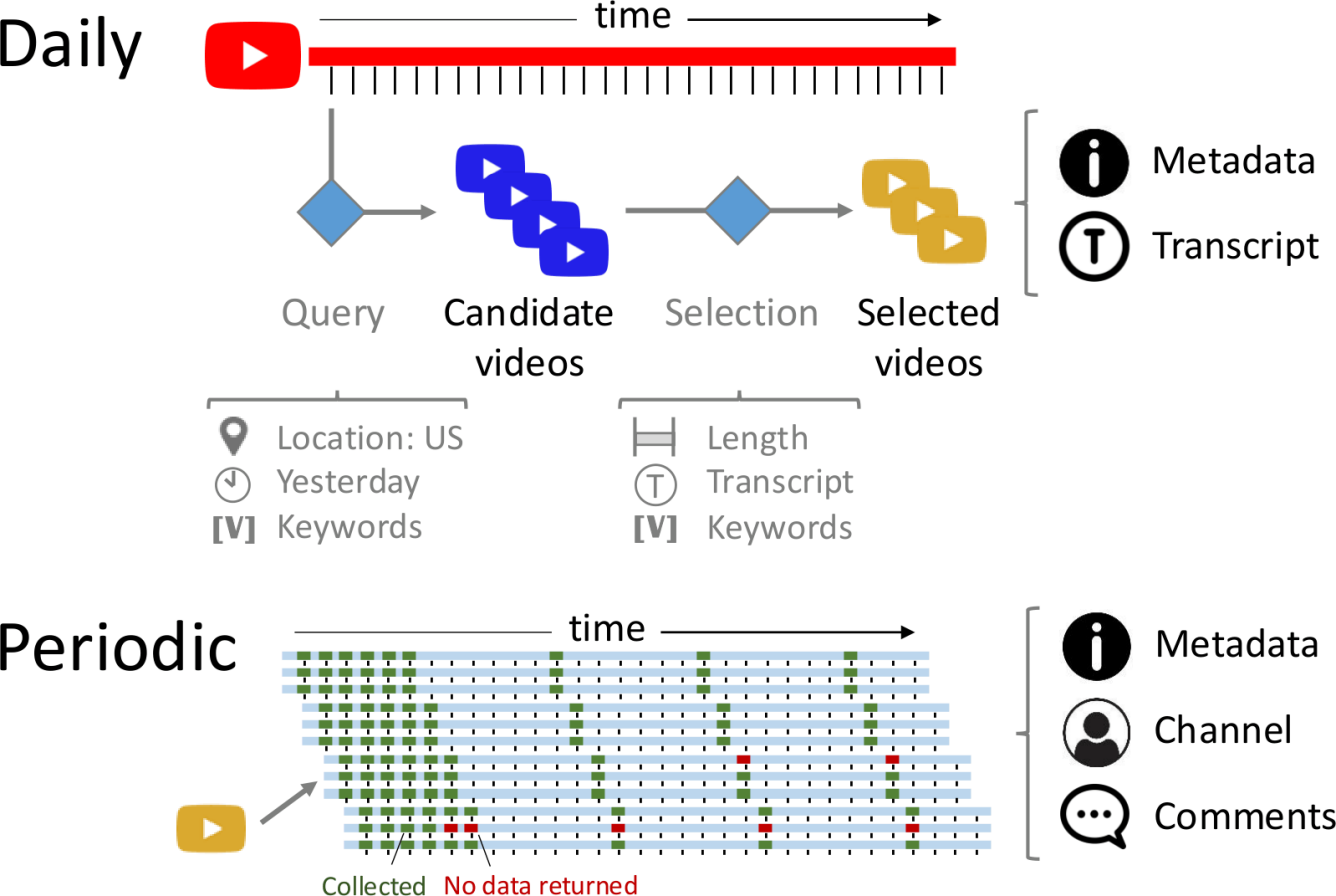
Data collection pipeline overview: the daily collection and video selection (top) and periodic collection (bottom).

The collection used in this study was created using a double-phase approach outlined in Figure 1. We adopt a “streaming” approach, wherein every day the collector takes 2 actions: it first uses a list of relevant keywords to search for videos published in the previous day and selects the videos that pass several filters for inclusion in the dataset, and then it periodically recollects the metadata about the selected videos.

Specifically, we use the YouTube Search application programming interface (API) [30] to query for videos matching the following keyword list: *vaccine*, *vaccination*, *novax*, *provax*, *antivaxx*, *antivaxxer* (guided by previous work [4,19,31,32]). We restrict the time of collection to the previous day, the location to the “US,” and the language to English. The results are sorted by relevance, and we save the top 500. Next, we use the YouTube Video API [33] to collect the metadata about the posted videos (note that it would be at most 1 day old), and the YouTube Transcript API library [34] to download their transcripts. These videos are then filtered through several selection steps to make sure they contain enough content for analysis and that they are relevant to the topic of vaccination. We require that the videos have a transcript, be at least 1 minute and at most 60 minutes in duration, and that at least 1 keyword from the query can be found in their title or transcript, or at least 3 keywords can be found in the transcript, if none exist in the title. This filter results in about 8.3% (7213/86,387) of the videos passing (mostly because so many videos are very short, the analysis of which we leave for future studies).

During the periodic phase of the collection, we requery the metadata information about the videos daily in order to compose a temporal picture of the audience’s engagement with them, as well as to capture any potential moderation actions taken by the platform. We perform this requerying every day for the first week after the video is initially retrieved, and following that, every week. After 3 months of collection, we consider whether in the past 2 time segments the counts of views, likes, or comments increase, and if they do not, we conclude the collection for those videos. During this collection, we also gather the 1000 most recent comments posted on that video using the comment Threads API, as well as the information about the poster of the video using the Channels API [35]. The final dataset spans 6 months (November 20, 2024, to May 20, 2025) and is composed of 7213 videos posted by 3872 unique channels, and their accompanying 911,219 comments. Of the 7213 videos initially collected, 302 were excluded due to missing channel-level metadata (eg, hidden subscriber counts) or engagement timestamps required for the regression models, resulting in a final analytical dataset of 6074 videos.

### Content Characterization

#### Videos

As the focus of this study is to monitor the extent to which vaccine-hesitant content is present in such data, we endeavor to classify the collected videos according to their stance on vaccination [36]. We begin by extracting excerpts from the video transcripts, of length 100 words, centered around the query keywords, which we then label (if more than 20 are found in a transcript, we randomly select 20). We then use the zero-shot classification via GPT-4o mini (OpenAI) to assign one of the following labels to each of the excerpts: “in favor of vaccination,” “against vaccination,” or “not in favor or against vaccination,” using the prompt shown in Section A.1 in Multimedia Appendix 1. This resulted in 55,310 annotated excerpts, an average of 8 per video. We validated this approach by manually annotating a random sample of 200 text segments. The agreement between the two authors of this study, computed on 60 segments, was near-perfect, at 96.7% (58/60) (Cohen κ=0.94), with 2 instances of disagreement between the “in favor of vaccine” and “not in favor or against vaccination” classes. The accuracy of the zero-shot classifier was estimated at 82.5% (165/200) (a marked improvement compared to 70% achieved by standard natural language processing tools evaluated in previous literature [37]). Per-class performance statistics can be seen in Table S1 in Multimedia Appendix 1. All of the errors were those with the middle label “neither” except for 4 instances where the disagreement was between the “against vaccination” and “in favor of vaccination,” suggesting that the main source of error may be due to the strength of the expressed stance. Next, we propagate these labels to the videos by considering the mean of the excerpt scores for each video, using the following encoding: −1=against vaccination, 1=in favor of vaccination, and 0=neither in favor nor against. For the ease of interpretation and analysis, we categorize the continuous scores into the following: [0.5, 1]: “strongly in favor” (SIF); (0, 0.5): “in favor” (IF); [0]: “neutral” (N); (−0.5, 0): “against” (A); and [−1.0, −0.5]: “strongly against” (SA) vaccines. This way, we were able to annotate 6074 videos posted by 3789 distinct users. The number of videos and channels in each category can be seen in the Results section.

#### Comments

To characterize the discourse surrounding the videos, we annotate a sample of comments using a staged pipeline. We begin by filtering our video dataset to focus on the most engaged content, retaining only videos with at least 10 comments. From this filtered set, we randomly sample up to 50 comments per video for annotation. The sampled comments for each video are processed in a single batched API call to an LLM (GPT-4o mini). To determine the comment agreement with the video, the model is provided with the video’s transcript and metadata as contextual reference (title, description, and transcript) and prompted to perform 2 analyses for each comment: first, to classify its agreement relative to the video’s main claims (“agree,” “disagree,” or “neutral/unrelated”), and second, to determine its sentiment (“positive,” “negative,” or “neutral”). The model is also instructed to provide a brief rationale for each classification. The specific prompt used for this task is detailed in Section A.2 in Multimedia Appendix 1. Further, the agreement label was annotated by the authors on a sample of 100 comments. Interannotator agreement, computed on 20 overlapping items, was *k*=0.66, indicating substantial agreement [38].

Comparing the labels to those produced by the classifier, 73 labels match exactly, 17 disagree with a “neutral/unrelated” label, and the remaining 10 have the opposite disagreement (“agree” vs “disagree”). Upon manual examination, we find that the classifier often either misinterprets the stance of a long transcript or misses a contextual connection between mentioned parties that is not explicitly mentioned in the text. As the accuracy of the classifier is 90% if the “neutral” label is not considered an error, we proceed with the analysis, while keeping in mind the tool’s limitations.

By using this zero-shot classification approach, we are able to scale our analysis to thousands of videos without relying on rigid keyword lists that might miss nuance. Conceptually, this method mimics human judgment regarding video stance and comment sentiment but applies it consistently across the entire dataset. This allows us to map the landscape of vaccine discourse with high granularity, ensuring that our distinction between “provaccine” and “vaccine-hesitant” content captures the true intent of the speaker rather than just the presence of specific medical terms.

#### Channel Characterization

To understand the source of the videos in our dataset, we characterize the channels using a two-stage process: (1) an automated, hierarchical classification of the video’s primary topic for each of the 3789 channels in the dataset, and (2) a manual annotation of the channel’s creator type and declared authority signals for a subset of 645 channels.

##### Hierarchical Topic Classification

A substantial proportion of channels (278/3789, 7.3%) have multiple topic assignments from YouTube’s metadata, reflecting the inherent multidimensionality of vaccine discourse [4,6]. To resolve these ambiguities for categorical analysis, we implement a 2-level hierarchical classification approach grounded in established cascade methodology [39]. First, following cascade principles [40,41], we use vaccine-related keywords to identify the broad topic category for each video, acknowledging that vaccine discourse cuts across multiple subjects [22]. Second, to resolve remaining multitopic conflicts, we apply a specificity prioritization rule: politics ≻ society ≻ health. This hierarchy is theoretically grounded in hypernym-hyponym relationships from taxonomy theory [39,42], ensuring that more specific and discriminative categories (hyponyms like politics) are retained over broader ones (hypernyms like society or health). This ensures that more specific, discriminative categories are preserved over broader ones, collapsing ambiguous assignments into a single primary topic while retaining critical information about content framing [39]. Details on the topic of co-occurrence statistics can be found in Table S2 in Multimedia Appendix 1.

##### Manual Annotation

For the second approach, we consider two samples of users: in the first are the channels that posted more than 3 videos in our dataset (339 channels), and the second sample includes channels that posted videos altogether totaling more than 10,000 views (451 channels). Combined, the two lists contain 645 unique channel IDs. The focus of the annotation is 2-fold: finding whether the channel associates itself with an organization or an individual, and what kinds of authoritative signals the channel projects. Such signals may include being a doctor, a news agency, or presenting expertise in the topics of health, science, or education. We also mark the channels that provide opinions, without mentioning other qualifications, and those centered around entertainment outside educational or scientific discourse. The page of each channel was examined and annotated manually. Seven channels were removed by the platform before they could be evaluated. Four were removed from further channel-related analysis due to a topical match, such as being about animals and mentioning animal vaccination.

By using both topical signals from YouTube and those from manual annotation, we provide a view of the authors of these videos both in terms of the narrative view of their presentation of the topic and in terms of their self-presentation as a possible authority on the subject.

### Engagement Metrics

#### Video-Level Analysis

To examine engagement patterns at the video level, we aggregated all metadata and computed normalized engagement rates. For each video *v*, the like ratio (LR) and comment ratio (CR) were defined as:

LR*_v_* = (video likeCount*_v_*)/(video viewCount*_v_*+1)

CR*_v_* = (video commentCount*_v_*)/(video viewCount*_v_*+1)

with extreme values clipped to the interval [0.001, 0.999] to satisfy the support of a beta regression. Stance labels—SIF, IF, N, A, and SA—were one-hot encoded, using neutral as the reference category. Channel-level covariates included the natural logarithm of the subscribers to the channels (LogSub) and the square root of the channels’ video counts (CHV):

LogSub*_v_* = ln(subscriberCount*_v_*)

CHV*_v_* = $\sqrt{videoCount}$

capturing audience reach and channel activity. Temporal exposure, measured in days since publication, controlled for the decay of engagement over time. These features served as predictors in our beta regression models of normalized engagement rates.

For clarity, we refer to the total counts of views, likes, and comments per video as raw interaction volume, and to the LR and CR as normalized engagement rates (interactions per view).

#### Comment-Level Analysis

We then characterized user engagement by studying the comments associated with each video. Our dataset initially consisted of 60,507 labeled individual comments. Videos with fewer than 5 annotated comments were excluded from the controversy analysis to ensure statistical reliability. Comments were categorized as agreeing, disagreeing (D), or N, and were used to compute entropy-based opinion diversity, or the controversy score (CS) [23,43]: CS_v_=*H*(C_A_, C_D_), where *H*(·) denotes Shannon entropy. Note that the measure portrays opinion heterogeneity, and not merely the existence of disagreement, making it possible that the comments are all in disagreement with the video, but homogeneously so, making the discussion not internally controversial.

### Statistical Analysis

#### Models

To statistically test our hypotheses regarding engagement, temporality, and discourse characteristics, we used a Bayesian framework. This approach provides a direct probabilistic interpretation of model parameters and full posterior distributions quantifying uncertainty. All models were specified using a consistent set of predictors to ensure comparability: the video’s stance label (with “neutral” as the reference category), the log-transformed subscriber count (LogSub), and the CHV.

We assigned weakly informative normal priors (*β_j_* ∼ Normal(0*,σ*^2^)) to all regression coefficients to regularize estimates and prevent overfitting.

#### Engagement Rate Models

To model the LR and CR, which are proportions bounded between 0 and 1, we specified a beta regression model for each, a standard approach for proportional outcomes, as it respects the bounded support of the data. For each video *i*, the engagement rate *y_i_* is assumed to follow a beta distribution:


(1) $$y_{i}∼Beta\;\left(\mu_{i}\cdot \kappa ,\;(1-\mu_{i})\cdot \kappa \right)$$

where the mean *µ_i_* is linked to the predictors via a logit link function:


(2) $$\mathrm{logit}(\mu_{i})=\beta_{0}+\beta_{\text{label}[i]}+\beta_{\text{LogSub}}\cdot {LogSub}_{i}+\beta_{\text{CHV}}\cdot {CHV}_{i}$$

The precision parameter *κ* was assigned a broad half-normal prior. The exponentiated coefficients are interpreted as odds ratios (ORs), representing the multiplicative change in odds of engagement per unit increase in the predictor.

#### Engagement Temporality Model

To model the speed of engagement, we predicted the number of days required to reach 90% of total engagement (*P*90). As this outcome is a positive integer count, we specified a *negative binomial regression model*, which is well-suited for overdispersed count data. The number of days *d_i_* for video *i* is modeled as:


(3) $$d_{i}∼\text{NegativeBinomial}(\mu_{i};\alpha )$$

where the mean *µ_i_* is linked to the predictors via a log link function, and *α* is the dispersion parameter:


(4) $$\log(\mu_{i})=\beta_{0}+\beta_{\text{label}[i]}+\beta_{\text{LogSub}}\cdot {\text{LogSub}}_{i}+\beta_{\text{CHV}}\cdot {\text{CHV}}_{i}$$

To control for endogeneity—specifically the mechanical correlation between low engagement volume and faster saturation—we fit a sensitivity specification adding log-transformed total engagement (*β*_Vol_ · log(TotalViews_*i*_)) to the linear predictor. The exponentiated coefficients from these models are interpreted as incidence rate ratios (IRRs), representing the multiplicative change in expected days to reach P90.

#### Discourse Characteristic Models

Finally, to analyze the nature of the comment section discourse, we fit two separate models.

*Disagreement model:* To predict the proportion of disagreeing comments (*prop*_disagree_), we used a beta regression model identical in structure to the engagement rate models (Equations 1 and 2).

*Controversy model:* To predict the CS, which is a continuous, unbounded variable, we specified a standard *Bayesian linear regression model* assuming a normal (Gaussian) likelihood. For video *i*, the CS is modeled as:


(5) $${\text{CS}}_{i}∼\text{Normal}(\mu_{i},\sigma^{2})$$

where the mean *µ_i_* is directly modeled as a linear combination of the predictors:


(6) $$\mu_{i}=\beta_{0}+\beta_{\text{label}[i]}+\beta_{\text{LogSub}}\cdot {\text{LogSub}}_{i}+\beta_{\text{CHV}}\cdot {\text{CHV}}_{i}$$

The coefficients (*β_j_*) from this model represent the direct additive change in the CS score.

Bayesian models allow us to move beyond simple averages to understand the probability of engagement. In the results that follow, we report ORs to measure the likelihood of interaction; a value greater than one indicates that a video is more likely to receive likes or comments than neutral content. We also report IRRs for our timing analysis; here, a value less than one signifies a “speedup,” meaning the video accumulates its audience and saturates its engagement potential more rapidly than the baseline.

Additionally, we validate findings under frequentist statistical frameworks and assess sensitivity to model specification. Results and details appear in Sections B-E in Multimedia Appendix 1.

### Ethical Considerations

This observational study analyzed publicly available YouTube content collected via the official YouTube Data API v3; it involved no direct interaction with human participants. Consequently, institutional review board (IRB) approval was deemed unnecessary, and the research complies entirely with YouTube’s Terms of Service. To protect user privacy, all individual usernames, user IDs, and identifying metadata associated with the comments were removed during data processing, and the comment text was deidentified. For transparency and reproducibility, examples of the public, high-profile YouTube channels analyzed in this study are listed in Multimedia Appendix 1. These channels represent public entities and major creators with no reasonable expectation of privacy regarding their broadcasted content.

## Results

### Videos

#### Overview

We begin by examining the user engagement with the videos in our dataset. As can be seen in Table 1, more videos have been classified as either “strongly in favor” or “in favor” of vaccination, although there are around 1500 videos in the “against” and “strongly against” categories.

**Table 1.** Video categories, score ranges, number of videos and channels in each category, and the proportion of videos to a channel. Note that the number of channels is presented per category and may overlap (the number of unique channels in the dataset is 3335).

Category	Score range	Videos, n	Channels, n	Videos/channel
Strongly in favor	[0.5, 1]	2492	1558	1.60
In favor	(0, 0.5)	1172	850	1.38
Neutral	[0]	996	796	1.25
Against	(–0.5, 0)	602	437	1.36
Strongly against	[–1.0, –0.5]	812	585	1.39

#### Extent of Engagement

**Figure 2.**
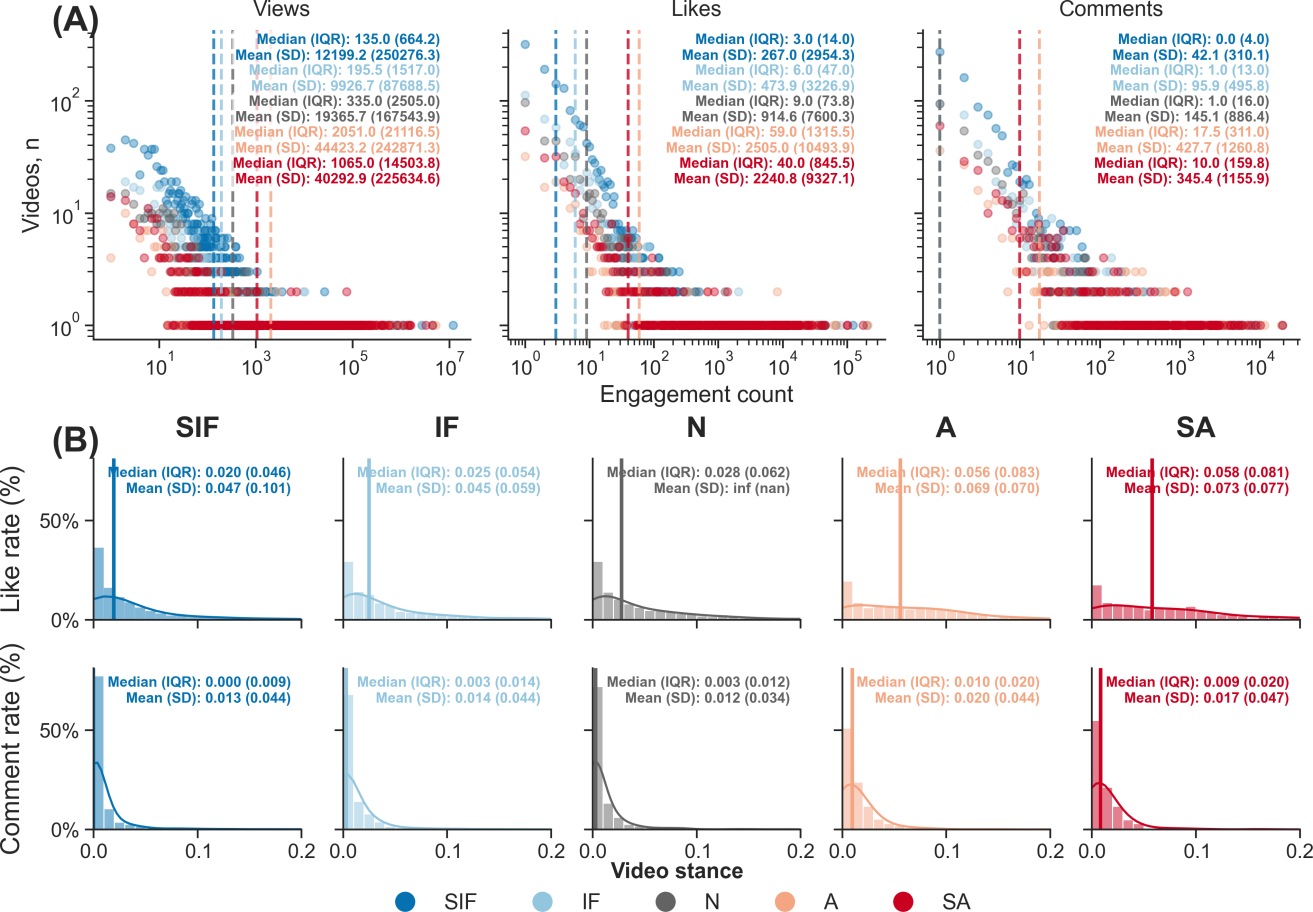
Distributions of video engagement by stance, shown in raw counts and normalized rates. (A) Log-log frequency plots for views, likes, and comments; dashed vertical lines indicate the median. The y-axis represents the number of videos. Vaccine-hesitant stances (“A” and “SA”) tend to have higher median engagement counts. (B) Histograms of normalized like ratio (LR) and comment ratio (CR), the y-axis represents the percentage of videos within each bin. Distributions for “A” and “SA” stances are shifted to the right, indicating a higher rate of engagement per view compared to provaccine (“SIF” and “IF”) and neutral (“N”) stances. A: against; IF: in favor; N: neutral; SA: strongly against; SIF: strongly in favor.

To establish the popularity of each video, we consider the last snapshot of its metadata that we have captured, which would be within a week of the end of the data collection period. Figure 2 shows the distributions of the number of views, likes, and comments for the videos in each of the 5 categories. The vertical lines signify the median value of the distribution, which is also shown in the upper right of each plot, as well as the mean. Because the distributions are heavy-tailed, the mean is affected by the few popular outliers and is thus sometimes several orders of magnitude larger than the median. Note that we are witnessing the videos at different times from their posting. Even though on average the videos labeled as “against” or “strongly against” are slightly “older” (mean 86 [SD 48] and mean 82 [SD 48], respectively) than those labeled as “in favor” or “strongly in favor” (mean 98 [SD 50] and mean 87 [SD 51], respectively), the standard deviations are so large that the effect of video age is negligible when comparing distributions within label categories.

Using all 3 metrics of engagement, we find that the videos in the A and SA categories receive much more attention than those IF and SIF categories. The difference in median views, for instance, differs by an order of magnitude from “strongly against” at 1065 (IQR 55-14,559) to “strongly in favor” at 135 (IQR 26-690). The views of “against” videos are even greater, at the median of 2051 (IQR 50-21,167). Further, 24.7% (615/2492) of the videos in the “strongly in favor” category received no likes at all, compared to 13.2% (107/812) of those in the “strongly against” category. The situation is even more dire for comments—a more strenuous action than a like—wherein the disparity is at 52.2% (1300/2492) compared to 27.5% (223/812), respectively. When normalized by views, these differences translate to substantial engagement asymmetries: vaccine-hesitant videos (“against” and “strongly against”) have median like ratio of 0.056 and 0.058 (ie, 5.6 and 5.8 likes per 100 views), respectively, compared to 0.020 for strongly in favor videos—a 2.8- to 2.9-fold engagement advantage. Combined raw interaction volume (likes+comments) yields approximately 10-fold higher engagement for vaccine-hesitant content (see Table S7 in Multimedia Appendix 1 for detailed comparison).

#### Statistical Analysis

**Figure 3.**
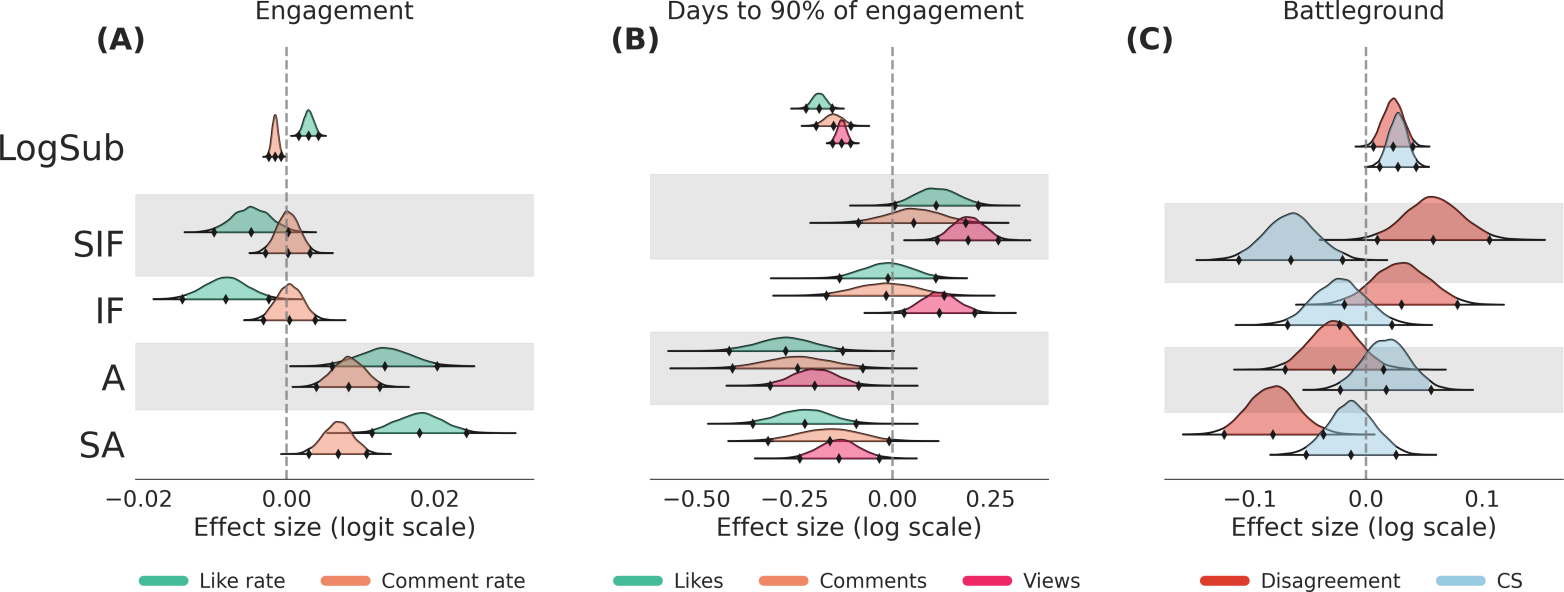
Posterior distributions of coefficients from Bayesian models of engagement and discourse. The figure summarizes the results of 3 sets of Bayesian regression models. Ridge plots show the posterior probability distribution for each predictor’s effect size on the logit (for rates) or log (for counts) scale. Horizontal bars represent the 94% highest density interval (HDI). A distribution to the right of the zero line indicates a positive association; to the left indicates a negative association. (A) Engagement: models predicting the like ratio and comment ratio. (B) Days to 90% of engagement: models predicting the number of days to reach 90% of total views, likes, or comments. A negative coefficient signifies a faster trajectory. (C) Discourse characteristics: models predicting the proportion of disagreeing comments and the controversy score (CS). The channel video counts (CHV) variable had no significant effect for all the models; it is not shown in the plots. Even when controlling for channel size, vaccine-hesitant stances are the strongest predictors of high normalized engagement rates and accelerated temporal saturation. A: against; IF: in favor; SA: strongly against; SIF: strongly in favor.

To provide a comprehensive statistical account of online vaccine discourse, we fit a series of Bayesian regression models predicting 3 key outcomes: engagement rates, engagement temporality, and discourse characteristics. The posterior distributions for the coefficients of each model are visualized in Figure 3. All comparisons are made relative to a neutral video stance. All model parameters converge (*R*≈1 for all variables).

Figure 3A displays the predictors for LR and CR. The results show a clear divergence in how audiences interact with pro- versus vaccine-hesitant content. For the like ratio model, we find strong evidence that vaccine-hesitant stances are associated with higher rates of likes compared to neutral content. The odds of a viewer liking a SA video are approximately 1.8% higher (OR 1.018, 94% highest density interval [HDI] 1.012-1.024), and 1.3% higher for an A video (OR 1.013, 94% HDI 1.007-1.020). The credible intervals for both coefficients are entirely above 1.0, indicating high certainty in the positive effect. Conversely, the IF stance is associated with a statistically credible decrease in the odds of receiving a like, with the odds being 0.8% lower (OR 0.992, 94% HDI 0.986-0.997). The effect for SIF is more uncertain, with a credible interval that just touches an OR of 1.0 (94% HDI 0.991-1.000).

The comment ratio model indicates that vaccine-hesitant content is most effective at stimulating discussion. Both A and SA stances are associated with a small but certain increase in the odds of commenting, with ORs of 1.008 (94% HDI 1.004-1.012) and 1.007 (94% HDI 1.003-1.011), respectively. The effects for provaccine stances (SIF and IF) are centered precisely on an OR of 1.0, with credible intervals that broadly overlap 1.0, indicating their effect on the comment ratio is indistinguishable from that of neutral content. Across both models, the channel characteristics LogSub and CHV showed very small and uncertain effects. Collectively, these models provide quantitative evidence that vaccine-hesitant content is more successful at generating positive feedback (likes), while also being slightly more effective at stimulating discussion (comments).

To validate these findings under alternative statistical frameworks, we conducted robustness checks using logit-ordinary least squares regression with heteroskedasticity-consistent (HC3) standard errors (Section B in Multimedia Appendix 1).

These models statistically confirm that the “engagement advantage” of vaccine-hesitant content is not a random artifact. The data show that a user watching a vaccine-skeptic video is significantly more likely to click “like” or leave a comment than a user watching a provaccine video, regardless of the channel’s size. This suggests that hesitant narratives are inherently more effective at eliciting active feedback from their audience.

### Engagement Over Time

**Figure 4.**
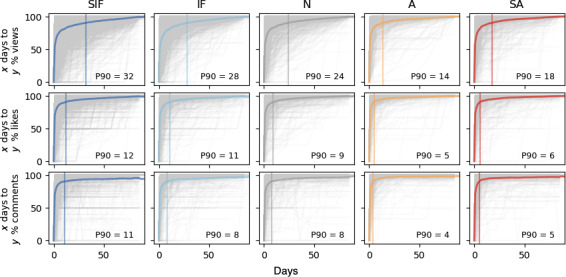
The engagement “trajectories” showing the percentage of views, likes, or comments (see row labels) the video achieves over days (x-axis), by label category (shown above each plot). The trajectories for each day are in light gray, and the average trajectory is in color. The vertical bar marks the number of days at which the average trajectory reaches 90% of the statistic. For likes and comments, videos are limited to those having more than 1 like or comment, respectively. Vaccine-hesitant content saturates significantly faster (median 18 days) than provaccine content (median 32 days), creating a narrow window for effective public health counter-messaging. A: against; IF: in favor; N: neutral; P90: 90% of total engagement; SA: strongly against; SIF: strongly in favor.

Next, we take advantage of the regular requerying of the engagement statistics to examine the temporal trend of the views, likes, and comments the video receives after its posting. Figure 4 shows the trajectories of views, likes, and comments of the videos in each of the label categories. For each statistic, we also compute an average trajectory line and show it in color. We also show (by the vertical line) the number of days at which the average trajectory reaches 90% (P90) of the statistic. For instance, videos labeled as “strongly in favor” on average reach 90% of their views at day 32 (IQR 18-64), whereas those labeled as “strongly against” reach it at day 18 (IQR 8-38).

Although it is not possible for views to decrease over time, it is possible for the number of likes or comments to decrease when a like is unselected or a comment is deleted. This introduces some noise, especially for those videos that have only one comment or like. For this reason, the analysis of the likes and comments includes only videos that had more than 1 like or comment (respectively) in our dataset. See Section H in Multimedia Appendix 1 for plots that include all videos, as well as the same statistics at thresholds 5 and 10.

Considering all of the engagement metrics, we find that the videos labeled as “against” or “strongly against” vaccination reach their peak engagement sooner than those in favor. Visually, the trend lines become sharper at the “knee” (where the distribution starts reaching a plateau). The pattern remains similar at thresholds 5 and 10 (see Section H in Multimedia Appendix 1), though the magnitude of the difference between “strongly in favor” and “strongly against” decreases in the case of comments, which are sparser than likes.

To quantitatively characterize the temporal saturation of engagements, we use a nonnegative binomial Bayesian model. The center panel of Figure 3B models the speed at which videos accumulate their audience, predicting the number of days required to reach 90% of total engagement (P90). The results from our Bayesian negative binomial models show that vaccine-hesitant content reaches its engagement peak significantly faster than neutral or provaccine content. For the trajectory of likes, videos with an A stance are expected to reach their P90 approximately 24% faster than neutral videos (IRR 0.76, 94% HDI 0.658-0.864). Similarly, SA videos are approximately 20% faster (IRR 0.80, 94% HDI 0.705-0.905).

In contrast, provaccine content exhibits a slower rate of engagement accumulation. SIF videos are predicted to take approximately 12% longer to reach their P90 peak (IRR 1.12, 94% HDI 1.016-1.242), with a credible interval entirely above 1.0. The effect for IF videos is indistinguishable from neutral content (IRR 0.99, 94% HDI 0.882-1.112). These patterns were consistent across the models for views, likes, and comments. Furthermore, we found a strong effect for channel size, with each unit increase in LogSub associated with a trajectory that is approximately 17% faster (IRR 0.83, 94% HDI 0.803-0.856), suggesting that videos from larger channels accumulate their engagement more quickly.

To address endogeneity of temporal saturation, we conducted a sensitivity analysis by refitting the negative binomial P90 models with additional control for log-transformed total views. This specification tests whether stance effects on days-to-saturation reflect genuine timing asymmetries or merely mechanical confounding. The mechanism of potential confounding is straightforward: videos receiving fewer total likes or views will, by definition, reach a 90% engagement threshold sooner than videos with higher engagement ceilings. If stance predicts both engagement volume and timing, the observed temporal asymmetry could be entirely mechanical rather than reflecting genuine differences in how quickly vaccine-hesitant versus provaccine content accumulates engagement.

The controlled models substantially attenuated the stance effects on P90 days. For “strongly against” videos, IRR for temporal saturation decreased from 0.56 (44% faster saturation; 94% HDI 0.50-0.62) in the uncontrolled baseline model to 0.80 (20% faster saturation; 94% HDI 0.70-0.90) after controlling for log(TotalViews). This 24 percentage point reduction represents substantial confounding. The control variable itself had a positive coefficient (IRR 1.042, 94% HDI 1.021-1.061), meaning that videos accumulating more total engagement reached P90 more slowly, consistent with a pattern of sustained engagement growth rather than front-loaded spikes. Approximately 54% of the uncontrolled 44% effect appears attributable to mechanical confounding, while 46% persists as a genuine timing advantage. This controlled estimate of 20% faster saturation for vaccine-hesitant content represents a more conservative but still substantial temporal asymmetry, indicating that these videos not only receive less total engagement but also concentrate their engagement into a shorter time window relative to their final engagement level. All parameters achieved acceptable convergence (*R* <1.01). Detailed results are shown in Section G in Multimedia Appendix 1.

In summary, we find that vaccine-hesitant content has a “speed advantage.” By raw timing, while provaccine content accumulates its audience gradually over a month (32 days to P90), vaccine-hesitant content experiences a concentrated burst of activity, saturating within 18 days (44% faster). However, a portion of this temporal advantage reflects mechanical confounding: videos with lower total engagement reach absolute thresholds sooner by definition. Sensitivity analysis controlling for log-transformed total engagement attenuates the effect from 44% to 20% faster saturation, indicating that approximately 54% of the raw temporal advantage reflects mechanical confounding while 46% persists as genuine temporal compression. This means vaccine-hesitant videos not only receive lower total engagement but also concentrate what engagement they receive into a narrower time window relative to their eventual engagement ceiling. Both the 44% practical effect and the 20% controlled effect have policy relevance: practitioners face the 44% saturation speed; researchers identify a 20% genuine temporal compression. This rapid early-stage lifecycle implies that the digital footprint of hesitant content is established quickly, suggesting that the window for effective counter-messaging or moderation is much narrower for this type of content.

In summary, the 18-day versus 32-day difference corresponds to a descriptive 44% faster saturation for vaccine-hesitant content, whereas the controlled IRR from the negative binomial model corresponds to a more conservative model-based estimate of 20% faster saturation after adjusting for total engagement volume.

### Comments

Beyond the quantity of audience interaction, we aimed to understand its nature. To this end, we classified comment stances relative to the video’s own stance, analyzing the results in aggregate, by channel topic, and through the CS.

**Figure 5.**
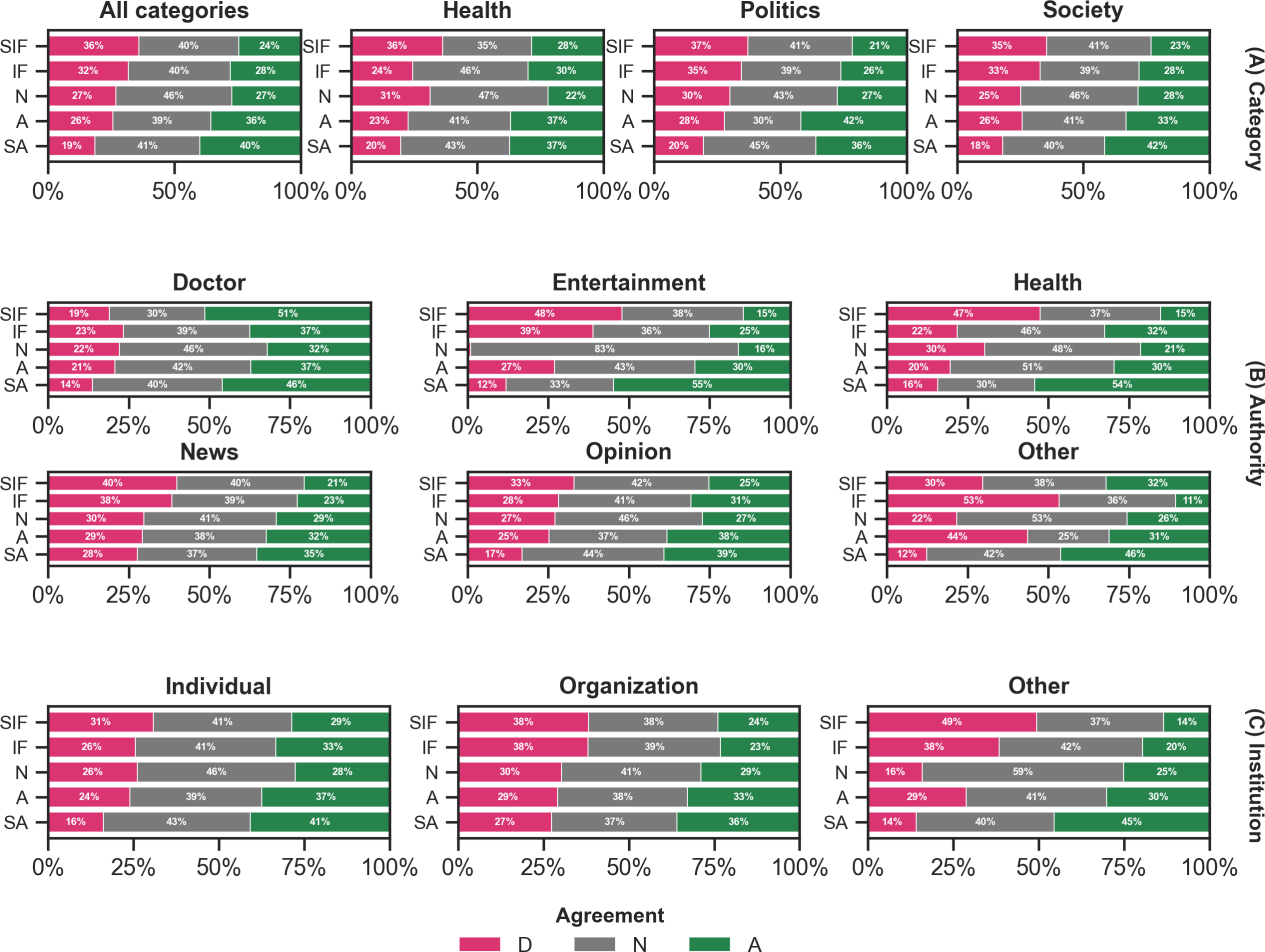
Comment agreement proportion by video stance, faceted by channel characteristics. The figure displays the normalized proportion of comment stances (D: disagree; N: neutral; A: agree) for videos, broken down by various channel categorizations. Each horizontal bar shows the proportion of comment types for a specific video stance (SIF: strongly in favor; IF: in favor; N: neutral; A: against; SA: strongly against) within that category. (A) Panels show distributions for channels grouped by their primary content category (eg, health and politics). (B) Panels show distributions for channels grouped by their declared authority (eg, doctor and news). (C) Panels show distributions for channels grouped by their institution type (individual, organization, other). Vaccine-hesitant videos function as confirmatory “echo chambers” (high agreement), while provaccine health videos serve as “battlegrounds” characterized by high levels of disagreement.

The aggregate results, shown in the “all categories” plot in Figure 5A, show that videos with SA and A stances are more likely to foster echo chambers; their comment sections are dominated by agreement, with 39.9% (6184/15,508) and 35.6% (6055/17,030) of comments agreeing, respectively (with only 18.6% (2889/15,508) and 25.7% (4379/17,030) disagreeing). In contrast, videos with SIF and IF stances attract significant contention, with higher disagreement rates of 35.9% (2751/7650) and 31.7% (2362/7440) and lower agreement rates of 24.4% (1870/7650) and 27.8% (2072/7440). This suggests that while vaccine-hesitant content fosters confirmatory engagement, provaccine content often serves as a “battleground” for public debate. Disaggregating by authority, we see that the “battleground” effect is most intense in health and entertainment (eg, 47.4% [138/291] disagreement on SIF health videos), while the knowledge topics (like doctor) appear more balanced, with “strongly in favor” videos attracting the highest rate of agreement (422/820, 51.5%) for any provaccine content.

**Figure 6.**
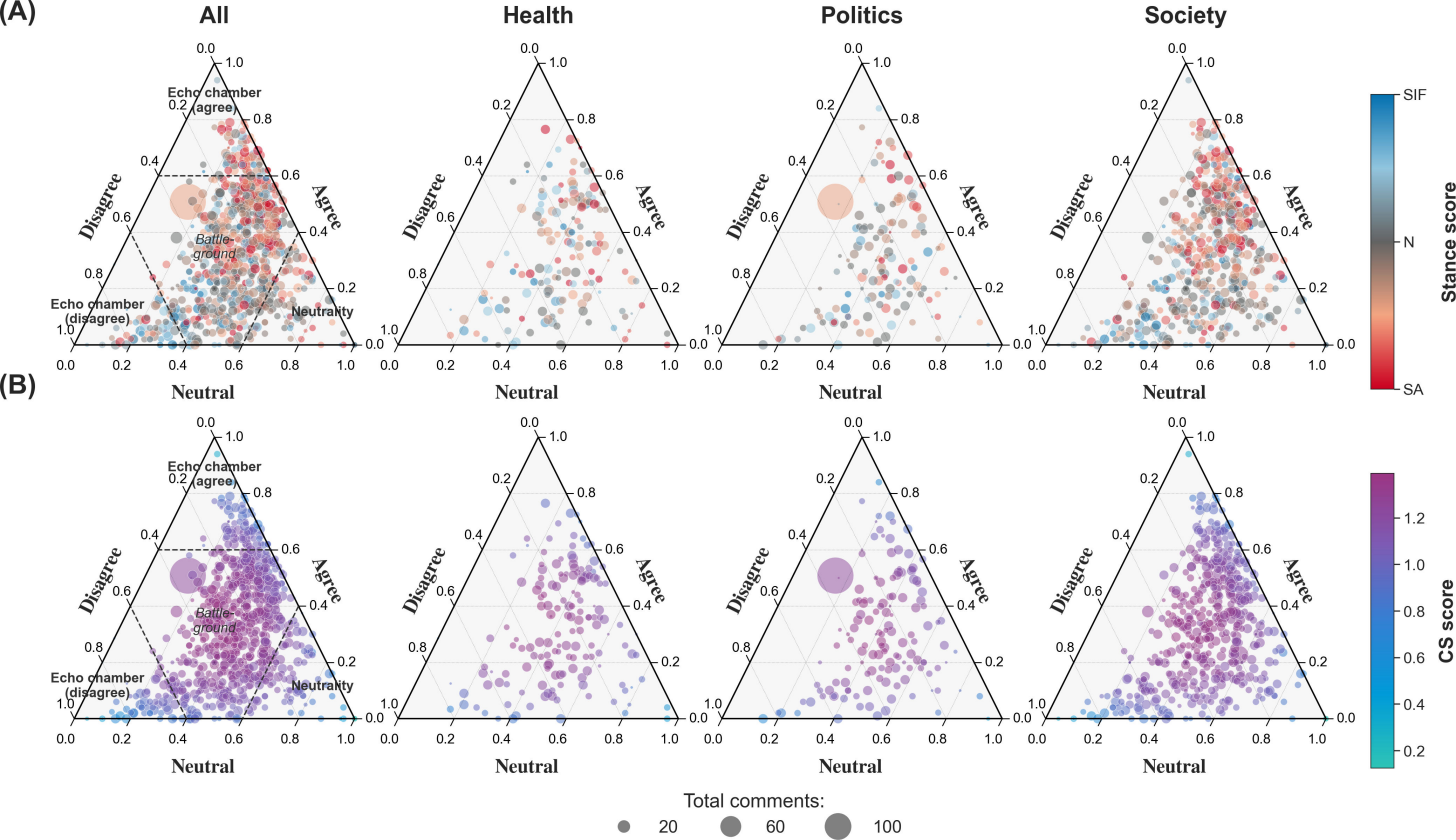
Visualizing discourse patterns through comment agreement distributions. This figure presents 3 perspectives on how audience agreement varies by video stance and topic. (A) A ternary plot visualizes each individual video’s discourse pattern, colored by its own stance (red=strongly against, blue=strongly in favor). The top corner represents high agreement (>60%), the bottom-left corner represents high disagreement (>60%), and the bottom-right represents neutrality. Dashed lines demarcate “echo chambers” (areas where a single stance dominates >60% of comments). Vaccine-hesitant videos (red) cluster heavily in the top “agree” echo chamber, particularly in society and politics. Provaccine videos (blue) are dispersed toward the bottom-left “disagree” corner and the center. N: neutral; SA: strongly against; SIF: strongly in favor. (B) The same distribution colored by controversy score (CS). Deep purple indicates high controversy (high entropy), corresponding to the “battleground” region in the center of the triangle where no single sentiment dominates. Bright teal indicates low controversy, aligning with the echo chambers at the corners. The platform simultaneously hosts an insulated agreement and intense public contention, mediated by the video’s stance.

These patterns are further explored in the ternary plots of Figure 6A, which visualize the distribution of individual videos in the tree level of agreement. Vaccine-hesitant videos (colored red and orange) mostly cluster in the top “agree” corner, providing strong visual evidence of echo chambers, particularly in the politics and society topics. Provaccine videos (colored blue) are distributed more broadly, with a significant concentration extending toward the bottom-left “disagree” corner and into the central battleground region, confirming their role as contentious battlegrounds.

Building on these observed patterns, we then colored the same distribution by the CS to quantify the level of contention (Figure 6B). The results confirm that the CS effectively captures the distinction between echo chambers and battlegrounds. The lowest controversy scores (bright teal) are heavily concentrated in the corners of the plots, particularly the top “agree” corner, aligning with the confirmatory echo chambers identified in the vaccine-hesitant videos. In contrast, the highest controversy scores (deep purple) are found in the center of the triangle, corresponding to the “battleground” videos that attract a chaotic mix of agreeing, disagreeing, and neutral comments. By comparing the topic-specific panels, we can see that the health topic contains a prominent cluster of high-controversy (purple) videos in its center, quantitatively confirming it is the most contentious domain. This shows that the CS metric captures the observed discourse patterns, mapping high consensus to low controversy and high conversational diversity to high controversy.

Figure 3C analyzes the nature of the comment section itself by modeling the proportion of disagreement and the CS. The results statistically confirm our “battleground vs echo chamber” hypothesis with quantitative evidence. In the *disagreement model*, provaccine stances are associated with a higher likelihood of attracting dissent. Compared to neutral videos, a SIF stance increases the odds of a comment disagreeing by approximately 5% (OR 1.05, 94% HDI 1.003-1.094). In contrast, a SA stance is associated with a decrease in the odds of disagreement by about 7% (OR 0.93, 94% HDI 0.898-0.972), with its credible interval falling entirely below 1.0. This provides strong statistical evidence that SA videos foster more agreeable, echo-chamber-like environments.

The *controversy score model* further clarifies this dynamic. The SIF stance is associated with a statistically credible decrease in the CS of approximately 0.075 units (94% HDI –0.119 to –0.033) compared to neutral content. At first glance, this seems counterintuitive. However, it suggests that while SIF videos attract more direct disagreement (a 2-way debate), neutral videos foster a more chaotic and unpredictable mix of “agree,” “disagree,” and “neutral” comments. The CS metric captures entropy, not just disagreement: perfectly balanced agree/disagree comments have lower CS than chaotic agree/disagree/neutral mixtures. Thus, more focused contention (SIF videos) can have lower CS than less predictable patterns. The effects of other stances (IF, A, SA) on the CS are uncertain, with credible intervals that all broadly overlap zero. Across both models, channel size (LogSub) has a significant positive effect, indicating that videos from larger channels tend to host both more disagreeable and more broadly controversial debates.

Our analysis delineates 2 distinct social environments coexisting on the platform. Vaccine-hesitant videos function largely as “echo chambers” where users reinforce each other’s views with high agreement. In contrast, provaccine videos—especially those from health institutions—function as “battlegrounds” characterized by debate and dissent. This distinction is crucial for interpretation, as it clarifies that high engagement numbers on provaccine content often reflect controversy and argument rather than consensus or support.

### Channels

Next, we show that the provaccine and vaccine-hesitant content largely originates from two distinct creator ecosystems. This section details the evidence for this sourcing asymmetry by examining the topical framing, authority signals, and institutional identity of the channels in our dataset.

To contextualize our findings, we first characterized the topical composition of the 7213 videos using the categories of the posting channel. Using a hierarchical resolution of YouTube’s metadata, we categorized videos into 3 primary topics: health (4027/6074, 66.3%), politics (918/6074, 15.1%), and society (628/6074, 10.3%). This hierarchical approach was necessary due to the prevalence of multitopic assignments in the original metadata (71.8% of videos), reflecting the inherently multidimensional nature of vaccine discourse. As shown in our subsequent analyses, this topical framing emerges as a significant moderator of engagement and comment discourse.

**Figure 7.**
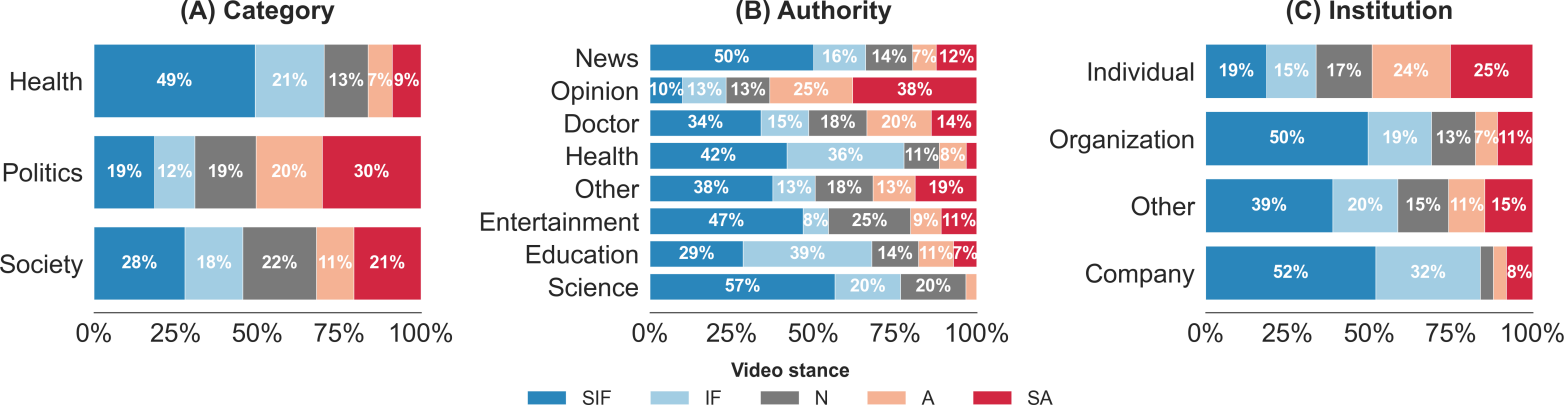
Share of videos posted by channels with certain labels per channel categories found in the metadata (A) and per authority (B) and institution (C) labels manually annotated on a subset of 645 channels. Provaccine discourse is dominated by institutional voices (news/health organizations), whereas vaccine-hesitant content is driven by individual creators, including self-identified medical doctors who bypass institutional constraints. A: against; IF: in favor; N: neutral; SA: strongly against; SIF: strongly in favor.

The distribution of video stances across these channel characteristics is presented in Figure 7. By topic (Figure 7A), health-related videos are slightly more provaccine (49.3% [1987/4027] “strongly in favor” and 21% [847/4027] “in favor”), whereas politics and society topics show more balanced distributions. This suggests health-framed discussions may attract greater institutional participation, while politics and society frames host a more diverse set of creator viewpoints.

This pattern is further intricate when considering the authority of a channel’s signals extracted via manual annotation (Figure 7B). Channels identified as news or about health predominantly post content in favor of vaccination, with 66% (870/1318) and 77.7% (237/305) falling into the combined “strongly in favor” and “in favor” categories, and with minimal representation in the strongly against category. In contrast, channels run by (self-declared) doctors post content both in support and against vaccination. Finally, entertainment and opinion channels display the most heterogeneous distributions, including significant portions of antivaccine content, suggesting these creators serve diverse audiences, often without the institutional constraints that guide health or news organizations.

Figure 7C shows a clear distinction between organizational and individual creators. Organizations are more represented in provaccine content production (1103/1598, 69%) of their videos fall in the “strongly in favor” and “in favor” categories combined, versus only 10.8% (172/1598) in “strongly against,” whereas individual creators exhibit greater stance diversity. Only 18.6% (132/708) of videos from individual creators are “strongly in favor,” while 25.1% (178/708) are “strongly against,” pointing to the need for engagement with individual posters to tackle such content.

These distributional patterns are reflected in the composition of the most prolific channels in our dataset (Section I in Multimedia Appendix 1). The 10 channels that posted the most videos are responsible for just 5% of the total volume (Gini=0.39), indicating that the content is not dominated by a few voices. This list includes a mix of news organizations, such as CTV and 9NEWS, which primarily post provaccine content, alongside individual creators like a self-described doctor or a showman, who are significant contributors to the vaccine-hesitant discourse. This mix illustrates the broader patterns in action: news media are an important contributor of vaccine-related content, but influencers and alternative voices have a strong and countervailing presence.

Finally, we investigated how the source of a video impacts audience discourse by analyzing comment agreement patterns across these channel types (Figure 5B,C). The results show that the nature of the creator significantly shapes the comment section’s dynamics. Within the Authority categorization, for instance, health channels exhibit the most polarized reactions, fostering strong echo chambers for “strongly against” videos (82% agreement) while their “strongly in favor” videos become “battlegrounds” (50% disagreement). When categorized by institution, a clear distinction appears: videos from organizations tend to generate stronger echo chambers, especially for vaccine-hesitant content, while individual creators host more balanced and contentious conversations across the board.

In summary, the analysis of channel characteristics shows that provaccine and vaccine-hesitant content largely originates from two distinct creator ecosystems. The provaccine discourse is predominantly driven by organizations, particularly news and health institutions, which reach cohesive audiences. In parallel, the vaccine-hesitant discourse is more strongly represented by a diverse array of individual creators, who foster more contentious and heterogeneous comment sections. This fundamental divergence in sourcing is crucial for understanding why these two types of content exhibit such different engagement dynamics and foster such different online communities.

### Moderation

**Figure 8.**
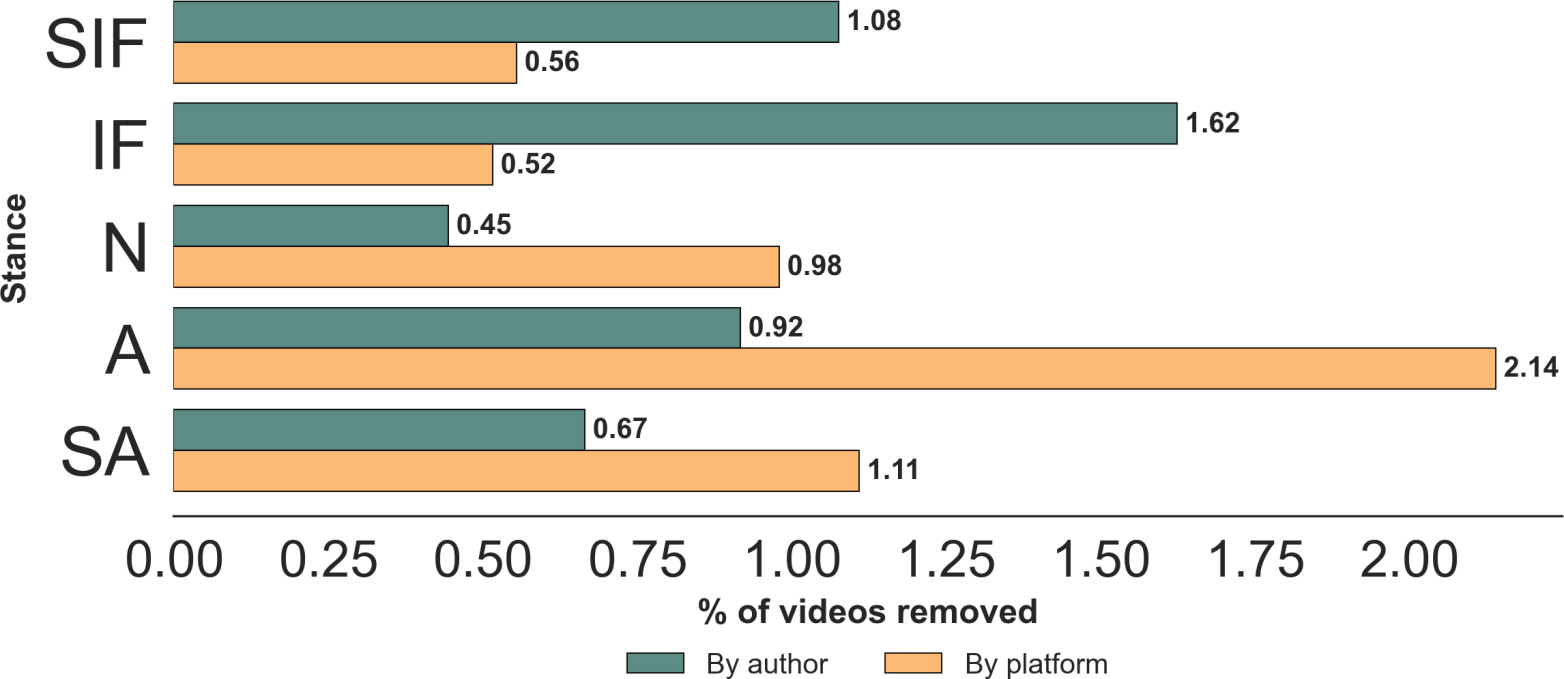
Percentage of videos in each stance that have been removed. Bars distinguish between videos made private or deleted by the uploader (by author) and those taken down for guideline violations (by platform). A: against; IF: in favor; N: neutral; SA: strongly against; SIF: strongly in favor.

In the course of the collection, 243 videos at some point failed to return metadata results during the periodic recollection efforts. We manually examined the errors the YouTube interface provided for these, and found that for 116, the platform simply tells the users that the “This video isn’t available anymore,” 66 have been made private by the uploader, 50 were unavailable due to the posting account having been terminated by the platform, 3 were removed by the uploader (a message distinct from the video simply being unavailable), 6 were removed due to violating YouTube’s Community Guidelines or Terms of Services, one because the uploader has closed their YouTube account, and one because of a copyright claim. We consider the video disappearances attributed to the author separately from all others (which we attribute to the platform), and find the rates shown in Figure 8. We find that the removals by the author of the video happen more for “strongly in favor” and “in favor” videos, whereas videos labeled as “against” or “strongly against” were more likely to be removed by the platform.

Considering the error codes more closely, we find that all videos removed due to violating YouTube’s Community Guidelines were either labeled as “against” or “strongly against.” On the other hand, videos that belonged to channels terminated by the platform span all 5 categories. Whenever the video was made private by its author, the label is more likely to be “in favor” or “strongly in favor” (77.3% [51/66] of such videos) than “against” or “strongly against” (9/66, 13.6%). Thus, we find a variety of reasons for the disappearance of videos, with some evidence of the platform’s policies affecting more the vaccine-skeptic side when it comes to sanctioning content, but not necessarily the accounts themselves.

The median time of the video disappearing is 14.4 (IQR 4.9-35.8) days from its posting, meaning that half of the videos were taken down within that time. However, many remained until almost the end of our 3-month collection period, pointing to a low but constant level of moderation across time. Since most of the interactions with the video happen within the first few weeks of a video’s posting, this policy likely fails to stem the majority of the video’s impact.

Upon the examination of the metadata collected for the videos of accounts terminated by the platform (as the videos themselves were not available at the time of the analysis), we find that they largely consist of vaccine-skeptic, anti-establishment content, as well as likely semiautomated channels that repost news snippets. Those labeled as “in favor” of vaccines sometimes use sensationalized language, such as “The truth might SHOCK you!” (such phrases are often considered “clickbait” to attract an audience). Some also engage in “keyword stuffing” by including many keywords in the description of the video to increase the chance of attaining a higher rank during a search. Yet other accounts mention vaccination in the context of providing other information, such as a channel promoting US visas. Unfortunately, beyond the error message, we cannot obtain any additional information on why these accounts might have been removed; however, the above observations exemplify the low-quality content that public health institutions compete with on this platform. The last engagement information our data collection captured for these removed videos reflects the overall trend of videos against vaccination, showing more engagement than those in favor (see Section J in Multimedia Appendix 1).

## Discussion

### Context

Prior YouTube vaccine studies have shown that vaccine-hesitant content receives more engagement than provaccine ones [10,11,44]. However, these snapshot-based analyses reveal only an engagement gap, leaving unanswered when and how fast this gap widens. By tracking engagement longitudinally, we document a previously unquantified temporal asymmetry: vaccine-hesitant videos saturate engagement significantly faster than provaccine content (44% faster in raw timing; 20% faster in model-based estimates that control for engagement volume). Furthermore, we challenge the assumption that polarization is symmetric [18,45]. Our comment analysis demonstrates that vaccine-hesitant videos function as agreeable echo chambers [23,46], whereas provaccine videos act as contentious battlegrounds. This suggests the platform hosts two distinct discourse architectures simultaneously, mediated by content stance.

Importantly, our analysis is purely observational: we do not observe recommendation paths or run interventions, and therefore we cannot identify causal effects of YouTube’s ranking or recommendation systems; we interpret our results as patterns of realized engagement rather than causal impacts of specific algorithms.

### Engagement Asymmetry and the Temporal Window for Intervention

By quantifying engagement dynamics, we observe that vaccine-hesitant videos attract substantially higher normalized engagement rates (2.8 to 2.9× higher) and reach saturation significantly faster than provaccine content. This temporal dimension suggests that vaccine-hesitant content gains traction not through sustained growth, but through rapid early-stage saturation. While the observed speed advantage (44% faster descriptively) is partly driven by lower engagement ceilings, a genuine temporal compression persists even after controlling for this confounding (20% faster after controlling for total engagement). This distinction between threshold-crossing speed and engagement velocity deserves deeper attention.

The decomposition of this temporal asymmetry shows distinct audience dynamics. The portion of the speedup attributable to confounding indicates that vaccine-hesitant audiences are smaller but more efficient: they mobilize quickly from a self-selected pool, producing a “peak-and-plateau” dynamic characteristic of viral misinformation [47]. Unlike provaccine content, which accumulates engagement incrementally as it diffuses through heterogeneous networks, vaccine-hesitant content exhibits the rapid consolidation typical of echo chambers [18,48]. This aligns with our comment-level findings: the structural homogeneity of the vaccine-skeptic audience facilitates rapid, confirmatory engagement, while the heterogeneity of the provaccine audience results in slower, contentious accumulation.

These findings identify a mechanism-specific temporal signature. Prior work established that false news spreads faster [6,15], but this study suggests that the temporal advantage operates through distinct audience structures: concentrated early-stage mobilization of in-groups versus distributed, delayed expansion into out-groups. From a policy perspective, this mechanism suggests that the critical window for intervention is not merely short (as commonly noted [6,49]) but compressed into a narrow audience demographic, concentrated in the initial 2 weeks post-upload.

### Implications for Intervention and Governance

The policy implications of this rapid saturation are 2-fold. First, the 18-day saturation window implies that practitioners must adopt real-time monitoring, as the majority of a video’s impact crystallizes faster than traditional fact-checking cycles can respond. Second, since moderation actions in our dataset occurred with a median delay of 14.4 (IQR 4.9-35.8) days [50,51], reactive content removal often arrives after the audience has already been saturated.

Therefore, effective intervention must target early audience composition rather than relying on post hoc removal. These empirical patterns motivate, but do not themselves test, potential intervention strategies. Conceptually, prebunking approaches that inoculate audiences against misinformation narratives [52-54] are particularly relevant here as they work before the rapid saturation occurs. Furthermore, saturated content is potentially amplified via cross-platform diffusion [55]. Platforms should consider proactive algorithmic demotion of borderline content or the elevation of credible sources during the first 2 weeks of a video’s lifecycle [56,57]. Rather than removing vaccine-hesitant content outright—a strategy fraught with free-speech concerns and often ineffective after saturation [58,59]—platforms could implement ranking systems that elevate credible sources in recommendation feeds, effectively competing for the attention of early audiences before echo chamber formation. Platform design choices that prioritize engagement metrics inadvertently amplify misinformation by selecting for emotionally resonant content [15,47]; reorienting algorithmic goals toward credibility rather than engagement represents a structural intervention with potential for large-scale impact [57,60].

While we found evidence of moderation, the median delay of 14.4 (IQR 4.9-35.8) days becomes critical when considered against the temporal dynamics we identified. Vaccine-hesitant videos concentrate their engagement—a pattern indicating rapid in-group mobilization characteristic of echo chambers—within approximately 18 days. Since moderation occurs 14.4 (IQR 4.9-35.8) days post-upload on median, content removal actions occur after the most intense period of audience concentration has already occurred, a finding that supports recent work on delayed moderation [50,51].

Given the complexities of content removal [58,59], one possible policy intervention could be the proactive algorithmic amplification of content from verified health authorities, especially during the early days of a video’s lifecycle [56,57,61]. Platform design choices that prioritize engagement can inadvertently amplify misinformation [15,47]; therefore, strategic interventions to elevate credible sources could significantly improve the health information environment without resorting to widespread censorship [60].

Our findings are consistent with these concerns, but we cannot speak directly about recommendation-system mechanisms. We therefore present these governance implications as hypotheses and directions for future experimental and audit studies, rather than as causal claims about the effects of specific policy levers.

### Discourse Patterns: The Role of Creators and Thematic Framing

According to our findings, online vaccine discourse is likely driven by two fundamentally different creator ecosystems. Provaccine content is predominantly produced by organizations (news and health institutions), whereas individual creators dominate the vaccine-hesitant space. This sourcing divergence is associated with different discourse patterns we observed: institutional videos tend to become “battlegrounds” of disagreement, while individual creators host “echo chambers” of agreement.

This dynamic may also be further modulated by thematic framing. Health-categorized videos, which attract the highest institutional participation, become the most intense sites of controversy [62,63]. In contrast, content framed around politics and society allows individual creators to cultivate highly agreeable, insulated communities [48,64]. This suggests a fundamental tension: institutional voices attempt to maintain a medical frame, but the discourse persistently escapes into social and political contexts where individual voices claim equal standing. To bridge this gap, public health bodies should consider partnerships with credible individual creators—such as doctors who vlog—who can foster dialogue without triggering immediate polarization [24,65,66].

### Institutional Context and Future Policy Dynamics

It is important to situate these findings within the broader landscape of vaccine policy. Our data collection (November 2024 to May 2025) captures a moment of significant institutional uncertainty. The observed engagement asymmetry likely reflects not merely ambient polarization but the consequence of eroded trust, wherein health institutions themselves become sites of political contestation.

This raises theoretical questions for future research: Do engagement asymmetries intensify during periods of institutional distrust? Longitudinal monitoring across different policy contexts would clarify whether our findings represent a transient response to uncertainty or a structural feature of polarized health discourse. Additionally, without systematic auditing of recommendation systems, the degree to which these asymmetries reflect platform design versus audience-driven polarization remains an open question [67]. Comparative studies across platforms and jurisdictions would strengthen our understanding of these mechanisms.

### Limitations

Our study has several limitations that define the scope of our findings.

#### Exclusion of Short-Form Video

Our analysis is intentionally focused on substantive, long-form YouTube videos (1‐60 minutes) and excludes short-form content like YouTube Shorts. This decision was made because Shorts exhibit fundamentally different engagement dynamics, algorithmic promotion, and content characteristics (eg, lack of transcripts) that would confound our analysis of engagement trajectories and argumentative stances [68,69]. While this exclusion enhances the internal validity of our findings on deliberative content, it limits their generalizability to the short-form video ecosystem, which represents a growing segment of online media consumption. Given the highly condensed and viral nature of Shorts, we hypothesize that their inclusion would likely amplify the rapid engagement saturation trends observed here, potentially fostering an even faster cycle of misinformation spread. Future research should analyze this content format separately, using methods suited to its visual and algorithmic nature.

#### Other Exclusion Criteria

Besides excluding short-form videos, each step of our collection more precisely defines the scope of this study, and thus limits its generalizability to other scenarios, such as other topics, time periods, or countries. The keyword selection, for instance, may have excluded the content that refers to vaccination obliquely and focuses on mainstream medicine in general [70]. Further, the focus of the collection was to sample the posted videos, and it is likely that some viral videos were not included in the sample, diminishing the extent of the skewness of our estimates of engagement. Studies using complementary methodology would be necessary to explore other aspects of the YouTube ecosystem.

#### Temporal and Geographic Scope

Data collection (November 2024 – May 2025) coincided with a period of heightened institutional contestation in US health policy. Consequently, the observed engagement dynamics likely characterize periods of high uncertainty rather than stable institutional consensus. Furthermore, as vaccine hesitancy drivers vary globally [71], our findings are specific to the US-English context. Cross-cultural research is needed to determine if the “engagement advantage” of hesitancy is a universal platform feature or a byproduct of specific sociopolitical environments, such as the high political polarization found in the United States.

#### Scalable Content Analysis Trade-Offs

To enable a large-scale longitudinal analysis, we used an LLM-based approach for content classification. While our method achieved high accuracy (84%) and perfect interrater reliability, it has limitations. First, stance classification was based on averaging scores from text excerpts, a method that may not fully capture videos with evolving narratives or strategic ambiguity. Second, the classification of texts is made difficult by the presence of humor, sarcasm, hyperbole, and other rhetorical devices that are challenging for both automated and human annotators to agree on (although the overall performance of the LLM-based approach largely agreed with that of the human labelers). Third, our comment analysis, classifying stance as agree/disagree/neutral, captures the balance of opinion but not the qualitative nature of the discourse. It does not distinguish between constructive debate and toxic trolling, nor does it analyze conversational structures like reply threads, which are important dimensions of online deliberation [72,73]. These trade-offs between scale and depth offer clear avenues for future qualitative and computational research.

#### Sensitivity to Misclassification in Automated Labeling

Our main constructs rely on automated LLM-based labeling. Validation against human annotations indicates an accuracy of 82.5% for video stance (with almost all errors involving the neutral class rather than confusion between pro- and antivaccine positions) and ≈73% exact agreement for comment agreement, rising to ≈90% when “neutral/unrelated” is not counted as an error. These error rates imply that misclassification primarily attenuates differences by shifting items toward the neutral or mixed categories, rather than systematically flipping provaccine into vaccine-hesitant content or agreement into disagreement. Given that our core effects are large—for example, vaccine-hesitant videos receive 10-fold higher raw interaction volume, 2.8- to 2.9-fold higher per-view engagement rates, and reach 90% of their engagement 20% faster even after controlling for engagement volume—the directionality of our findings is robust to plausible levels of label noise. At the comment level, replacing up to 10%‐15% of labels with their opposite direction would reduce but not reverse the observed pattern. Vaccine-hesitant videos’ comment sections are dominated by agreement (eg, 40% agree vs 19% disagree), and provaccine videos attract higher disagreement (eg, 36% disagree vs 24% agree), so the classification uncertainty is more likely to bias our estimates toward conservative lower bounds than to generate spurious asymmetries.

### Conclusions

This longitudinal study provides systematic evidence that vaccine-hesitant content on YouTube benefits from a distinct engagement advantage: it attracts approximately 10-fold higher raw interaction volume (and 2.8- to 2.9-fold higher per-view engagement rates) relative to provaccine content and reaches audience saturation significantly faster (44% faster in descriptive medians and 20% faster in adjusted regression estimates) than provaccine content. We identify a specific temporal signature—an 18-day saturation window—driven by audience structure. Vaccine-hesitant videos mobilize homogeneous, self-selected in-groups that generate rapid, confirmatory “echo chambers,” whereas provaccine content accumulates engagement incrementally through contentious “battlegrounds” of debate.

The policy implications of this mechanism are direct. Because the median time to moderation (14.4 days) lags behind the rapid saturation of hesitant narratives, reactive content removal is structurally ill-suited to mitigate misinformation. An effective public health strategy must therefore shift from post hoc rebuttal to proactive intervention. Prebunking campaigns and algorithmic ranking adjustments that elevate credible sources during the critical first 2 weeks of a video’s lifecycle represent the most viable levers for competing with the rapid mobilization of hesitant communities.

While this study is limited to long-form English content and acknowledges the trade-offs inherent to scalable LLM classification, it establishes baseline temporal patterns essential for evidence-based policymaking. Future research must extend beyond single-platform dynamics to examine how recommendation algorithms specifically amplify these asymmetries and how content diffuses across platforms. Ultimately, navigating the complex digital health environment requires not just producing scientific knowledge, but understanding the precise temporal and structural mechanisms by which that knowledge competes for public attention.

## Supplementary material

10.2196/88519 Multimedia Appendix 1 Data preprocessing and classification, detailed statistical analysis, and top 10 channels’ description.

## Abbreviations

A: against vaccination stance
API: application programming interface
CHV: channel video counts
CR: comment ratio
CS: controversy score
HC3: heteroskedasticity-consistent
HDI: highest density interval
IF: in favor of vaccination stance
IRB: institutional review board
IRR: incidence rate ratio
LLM: large language model
LR: like ratio
N: neutral to vaccination stance
OR: odds ratio
SA: strongly against vaccination stance
SIF: strongly in favor of vaccination stance
