# Supplementary material for: Engagement Trends in Online Vaccine Content: Longitudinal YouTube Study
Source: J Med Internet Res. 2026 Jun 19;28:e88519. doi: 10.2196/88519 (PMC13282043; doi:10.2196/88519)
Supplement: Multimedia Appendix 1 [file jmir-v28-e88519-s001.docx]

## Multimedia Appendix

## A. Data Preprocessing

We analyzed YouTube comments related to vaccine videos collected between November 20, 2024 and May 20, 2025.

**A.1 Prompt for Video Stance Labeling and Its Evaluation**

The prompt for the video classification to ChatGPT model gpt-4o-mini is as follows:

Your task is to determine whether the provided text is in favor of vaccination, against vaccination, or neither.

For the text, assign one of the following labels based on your assessment:

1. “in favor of vaccination” if the overall message is that vaccines are good and helpful and people should vaccinate. Alternatively, the text could quote or talk about somebody who is against vaccines, but if the text is against that person, the label should be “in favor of vaccination”.
2. “against vaccination” if the overall message is that vaccines are dangerous or harmful and people should not vaccinate or given a choice not to vaccinate. Alternatively, the text could quote or talk about somebody who is in favor of vaccines, but if the text is against that person, the label should be “against vaccination”.
3. “neither” if the text is not in favor or against vaccines.

Reply providing just the label. Here is the text: *[text]*

Table S1: Confusion tables, precision, recall, and F1 measure for each class for the video stance classification task.

| In favor of vaccination | | | | |  |
| --- | --- | --- | --- | --- | --- |
|  | Predicted | |  |  |  |
| Gold standard | T | F |  | Precision | 0.966 |
| T | 84 | 26 |  | Recall | 0.764 |
| F | 3 | 81 |  | F1 | 0.853 |
|  |  |  |  |  |  |
| Against vaccination | | | | |  |
|  | Predicted | |  |  |  |
| Gold standard | T | F |  | Precision | 0.864 |
| T | 19 | 3 |  | Recall | 0.764 |
| F | 3 | 146 |  | F1 | 0.811 |
|  |  |  |  |  |  |
| Neither | | | | |  |
|  | Predicted | |  |  |  |
| Gold standard | T | F |  | Precision | 0.681 |
| T | 62 | 29 |  | Recall | 0.969 |
| F | 29 | 103 |  | F1 | 0.800 |

**A.2 Prompt for Comment Agreement Labeling**

The prompt for the comments classification to ChatGPT model gpt-4o-mini is as follows:

You are an expert AI analyst. Your task is to analyze a batch of YouTube comments for a single video. You will be given the video’s context (title, description, transcript) and a list of comments, each with a unique ‘commentId‘.

For EACH comment in the batch, perform two analyses:

1. Classification against Video Claims: Determine if the comment ’Agrees’, ’Disagree’, or is ’Neutral/Unrelated’ to the video’s main claims.
2. Comment Sentiment Analysis: Independently, determine if the comment’s own sentiment is ’Positive’, ’Negative’, or ’Neutral’.

Here is the text: *[text]*

**A.3 Channel Characterization**

Table S2: Topic co-occurrence in YouTube metadata before hierarchical resolution. Cell (i,j) indicates the number of videos assigned to both topic category i and j in YouTube’s topicDetails.topicCategories metadata field. Approximately 71.83% of videos (3,996 items) received multiple simultaneous topic assignments. The hierarchical resolution applied cascade classification principles to assign each video to a single primary category: (1) domain-specific vaccine keywords anchored the primary semantic domain; (2) specificity prioritization (Politics ≻ Society ≻ Health) resolved conflicts. This process ensured that more informative, specific categorizations were retained while preserving the signal about vaccine discourse multidimensionality.

|  | Health | Politics | Society | Total |
| --- | --- | --- | --- | --- |
| Health-only | 947 | – | – | 947 |
| Politics-only | – | 2 | – | 2 |
| Society-only | – | – | 618 | 618 |
| Health + Politics | 37 | 37 | – | 37 |
| Health + Society | 3043 | – | 3043 | 3043 |
| Politics + Society | – | 916 | 916 | 916 |
| Health + Politics + Society | 37 | 37 | 37 | 37 |
| Total raw assignments | 4064 | 992 | 4614 | – |
| After hierarchical resolution | 947 | 955 | 3661 | 5563 |

**B. Logit-OLS Regression With HC3 Robust Standard Errors**

To complement the primary Bayesian Beta regression analysis, we fit ordinary least squares (OLS) regression models to logit-transformed engagement rates (Like Rate and Comment Rate) using heteroskedasticity-consistent HC3 standard errors. This frequentist approach provides independent validation of the engagement asymmetry findings. Frequentist estimates from this approach closely mirror Bayesian results and confirm the engagement asymmetry: vaccine-hesitant stances (A, SA) are associated with significantly higher engagement odds (OR = 1.31–1.52 for like rate; OR = 2.08–2.32 for comment rate), while pro-vaccine stances (SIF, IF) show reduced engagement odds (OR = 0.62–0.85 for like rate; OR = 0.43–0.68 for comment rate). These findings demonstrate that the engagement asymmetry is robust across Bayesian and frequentist specifications and is not an artifact of model choice.

**C. Robustness and Sensitivity Analyses**

To validate findings under alternative statistical frameworks and assess sensitivity to model specification, we conducted the following robustness checks:

**Frequentist Validation.** We re-estimated Like Rate and Comment Rate models using logit-OLS regression with heteroskedasticity-consistent (HC3) standard errors, a frequentist alternative to Beta regression. This approach tests whether findings depend on the choice of the Bayesian Beta regression framework. Coefficients were exponentiated to Odds Ratios (OR) for comparability with Bayesian estimates.

Table S3: Logit-OLS and Bayesian Beta Regression Results for Like Rate (LR). Comparison of frequentist and Bayesian estimates for the effect of video stance on normalized like rates (likes per view). Bayesian results report Odds Ratios (OR) with 94% Highest Density Intervals (HDI); OLS results report logit-scale coefficients converted to OR with 95% confidence intervals (CI). Neutral stance is the reference category (β = 0). All models include covariates for log subscriber count (LogSub) and channel video counts (CHV).

| Stance | Bayesian OR | Bayesian 94% HDI | | OLS OR |  | OLS 95% CI |
| --- | --- | --- | --- | --- | --- | --- |
| SIF | 0*.*995 | [0*.*985 | 1 005] | 0*.*62^∗∗∗^ | [0*.*46 | 0*.*84] |
| IF | 0*.*992^∗^ | [0*.*986 | 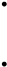0 997] | 0*.*85 | [0*.*63 | 1*.*14] |
| N (Reference) | 1*.*000 |  | — | 1*.*000 |  | — |
| A | 1*.*013^∗^ | [1*.*007 | 1*.*020] | 1*.*31 | [0*.*97 | 1*.*77] |
| SA | 1*.*018^∗∗^ | [1*.*012 | 1*.*024] | 1*.*52^∗^ | [1*.*08 | 2*.*13] |

94% HDI excludes zero (Bayesian); * p < 0.05 (OLS); ** p < 0.01 (OLS); *** p < 0.001 (OLS); Interpretation: Bayesian OR close to 1.0 indicate small effects on logit scale; OLS estimates are substantially larger because they compare logit-transformed rates. The Bayesian and OLS approaches yield consistent qualitative conclusions: vaccine-hesitant stances (A, SA) increase like rates, while pro-vaccine stances (SIF, IF) decrease or show no effect.

Table S4: Logit-OLS and Bayesian Beta Regression Results for Comment Rate (CR). Comparison of frequentist and Bayesian estimates for the effect of video stance on normalized comment rates (comments per view). Same format and interpretation as Table S1. The asymmetry is pronounced: vaccine-hesitant videos (A, SA) have 2.08–2.32 times higher odds of comments (OLS), while pro-vaccine videos (SIF, IF) show substantially lower odds (0.43–0.68).

| Stance | Bayesian OR | Bayesian 94% HDI | | OLS OR |  | OLS 95% CI |
| --- | --- | --- | --- | --- | --- | --- |
| SIF | 1*.*000 | [0*.*995 | 1*.*005] | 0*.*43^∗∗∗^ | [0*.*32 | 0*.*59] |
| IF | 1*.*000 | [0*.*999 | 1*.*001] | 0*.*68^∗^ | [0*.*50 | 0*.*93] |
| N (Reference) | 1*.*000 |  | — | 1*.*000 |  | — |
| A | 1*.*008^∗^ | [1*.*004 | 1*.*012] | 2*.*08^∗∗∗^ | [1*.*54 | 2*.*81] |
| SA | 1*.*007^∗^ | [1*.*003 | 1*.*011] | 2*.*32^∗∗∗^ | [1*.*72 | 3*.*13] |

94% HDI excludes zero (Bayesian); * p < 0.05 (OLS); *** p < 0.001 (OLS); Interpretation: Comment rate asymmetry is more pronounced than like rate: vaccine-hesitant stances show 2–2.3 times higher odds of comments, while pro-vaccine stances show 0.4–0.7 times lower odds. This indicates that vaccine-hesitant content is substantially more effective at stimulating discussion (comments), a more effortful action than likes.

**Threshold Sensitivity.** We re-estimated the Like Rate Bayesian Beta regression at progressively strict minimum engagement cutoffs (videos with ≥ 1, ≥ 5, ≥ 10 likes) to test whether findings depend on inclusion of minimally engaged videos or are driven by rare high-engagement outliers.

Table S5: Threshold Sensitivity Analysis: Like Rate Bayesian Beta Regression. Stance effects on Like Rate (OR) re-estimated at progressively strict minimum engagement thresholds. Videos are retained only if they have at least the specified number of likes (columns: 1, 5, or 10 likes minimum). All coefficients are Odds Ratios with 94% Highest Density Intervals. Neutral stance is the reference category.

Stance Threshold ≥ 1 Like Threshold ≥ 5 Likes Threshold ≥ 10 Likes

|  | OR | 94% HDI | OR | 94% HDI | OR | 94% HDI |
| --- | --- | --- | --- | --- | --- | --- |
| SIF | 0*.*995 | [0*.*985*,*1*.*005] | 1*.*001 | [0*.*987*,*1*.*015] | 1*.*008 | [0*.*989*,*1*.*029] |
| IF | 0*.*992 | [0*.*986*,*0*.*997] | 0*.*985 | [0*.*975*,*0*.*996] | 0*.*992 | [0*.*977*,*1*.*008] |
| N (Reference) | 1*.*000 | — | 1*.*000 | — | 1*.*000 | — |
| A | 1*.*013 | [1*.*007*,*1*.*020] | 1*.*011 | [1*.*003*,*1*.*019] | 1*.*014 | [1*.*002*,*1*.*027] |
| SA | 1*.*018 | [1*.*012*,*1*.*024] | 1*.*019 | [1*.*010*,*1*.*029] | 1*.*021 | [1*.*007*,*1*.*036] |

Interpretation: Stance effects on like rate are remarkably consistent across all engagement thresholds. The credible intervals overlap substantially across thresholds, demonstrating that the engagement asymmetry is a robust feature of the vaccine-related YouTube ecosystem. Even when restricting to videos with at least 10 likes, vaccine-hesitant content maintains its engagement advantage.

**D. Endogeneity Control: P90 Models With Engagement Volume**

To address potential mechanical confounding in temporal saturation estimates, we refitted the Negative Binomial P90 model with an additional control variable: log-transformed total engagement. This tests whether stance effects on days-to-saturation reflect genuine temporal compression or merely lower engagement volumes reaching thresholds sooner. The comparison shows the proportion of the raw effect attributable to confounding versus genuine temporal dynamics.

Table S6: Addressing Endogeneity in Temporal Saturation Analysis. Comparison of Negative Binomial regression models predicting days to 90% like accumulation (P90 Likes), with and without control for log-transformed total engagement. The uncontrolled model shows that vaccine-hesitant videos reach saturation 44% faster (IRR = 0.56); controlling for engagement volume reduces this to 20% faster (IRR = 0.80), indicating that approximately 54% of the temporal advantage reflects mechanical confounding (lower-engagement videos reach thresholds sooner by definition). The remaining 20% represents genuine temporal compression.

| Stance | Uncontrolled Model | |  | Controlled for Log(Total Likes) |
| --- | --- | --- | --- | --- |
|  | IRR | 94% HDI | IRR | 94% HDI |
| SIF | 1.112 | [1.016, 1.242] | 1.089 | [0.998, 1.195] |
| IF | 0.988 | [0.882, 1.112] | 1.008 | [0.916, 1.116] |
| N (Reference) | 1.000 | — | 1.000 | — |
| A | 0.760 | [0.658, 0.864] | 0.828 | [0.733, 0.939] |
| SA | 0.560 | [0.502, 0.623] | 0.798 | [0.705, 0.905] |
| Log(Total Likes) | — | — | 1.042 | [1.021, 1.061] |

IRR = Incidence Rate Ratio. Values < 1 indicate faster saturation (fewer days); > 1 indicate slower saturation. Interpretation: The control variable has a positive coefficient (IRR = 1.042), meaning videos with more total likes reach P90 more slowly, consistent with sustained engagement growth. For SA (Strongly Against) videos, the uncontrolled effect (IRR = 0.56, or 44% faster saturation) is substantially attenuated to a controlled effect of IRR = 0.80 (20% faster). This 24 percentage point reduction indicates that 54% of the raw temporal advantage reflects mechanical confounding, while 46% persists as genuine temporal compression. The controlled estimate of 20% is more conservative but still substantial, supporting the headline claim while acknowledging confounding bias.

**E. Bayesian Model Convergence Diagnostics**

The models were fitted using the No-U-Turn Sampler (NUTS), a variant of Markov Chain Monte Carlo (MCMC), as implemented in the PyMC library via the bambi interface. We ran four parallel chains, each with 1000 tuning steps and 2000 posterior samples. To ensure robust convergence and reliable posterior estimates, we confirmed that the potential scale reduction factor,$\hat{R}$, was approximately 1.0 for all parameters, indicating that all chains converged to the same target distribution. Additionally, we visually inspected trace plots to ensure the chains were well-mixed and stationary. These diagnostics ensure that reported posterior distributions and credible intervals are reliable and not artifacts of MCMC sampling issues.

**F. Engagement Quantification: Descriptive Statistics**

Table S7: Comprehensive Engagement Metrics: Raw Counts and Normalized Rates by Stance. Median and mean values for raw engagement (Views, Likes, Comments) and normalized rates (Like Rate = likes per view; Comment Rate = comments per view). The rightmost columns show fold differences relative to Neutral content, illustrating the magnitude of the engagement asymmetry.

| Stance Views | | | | | Likes | | | Comments | | Like Rate | Comment Rate |
| --- | --- | --- | --- | --- | --- | --- | --- | --- | --- | --- | --- |
|  | Median | Mean | | Median | | Mean | Median | | Mean | Median | Median |
| SIF | 135 | 12,199 | | 3 | | 267 | 0 | | 42 | 0.010 | 0.000 |
| IF | 196 | 9,927 | | 6 | | 474 | 1 | | 96 | 0.025 | 0.003 |
| N | 335 | 19,366 | | 9 | | 915 | 1 | | 147 | 0.028 | 0.003 |
| A | 2,051 | 44,423 | | 59 | | 2,505 | 17.5 | | 428 | 0.056 | 0.010 |
| SA | 1,065 | 40,293 | | 40 | | 2,241 | 10 | | 345 | 0.058 | 0.009 |
| Fold-Difference vs. Neutral (N): | | | | | |  |  | |  |  |  |
| SIF 0.40× | | | — | 0.33× | | — | — | | — | 0.71× | 0.0× |
| IF 0.58 × | | | — | 0.67× | | — | — | | — | 0.89× | 1.02× |
| A 6.12× | | | — | 6.56× | | — | — | | — | 2.00× | 3.31× |
| SA 3.18× | | | — | 4.44× | | — | — | | — | 2.08× | 2.85× |

Interpretation: Median views for vaccine-hesitant videos (A: 2,051; SA: 1,065) are roughly 8–15 times higher than strongly pro-vaccine videos (SIF: 135). Normalized engagement rates show even larger differences: vaccine-hesitant videos have 2.0–3.3 times higher like and comment rates than neutral videos.

**G. Temporal Quantification: Days to 90% Engagement Saturation (P90)**

Table S8: Temporal Dynamics: Median Days to Reach 90% of Total Engagement (P90). Descriptive statistics for the number of days required for each video to accumulate 90% of its total engagement, separately by engagement metric (Views, Likes, Comments) and stance category. P90 provides a direct measure of engagement saturation velocity: smaller values indicate faster audience buildup. These statistics complement the Negative Binomial regression results in the main text by showing absolute timing differences across stances.

Stance Views P90 Likes P90 Comments P90

Median (Q1, Q3) Median (Q1, Q3) Median (Q1, Q3)

| SIF | 32 days | (18, 64) | 30 days | (15, 58) | 29 days | (13, 56) |
| --- | --- | --- | --- | --- | --- | --- |
| IF | 29 days | (16, 61) | 27 days | (13, 55) | 26 days | (12, 53) |
| N (Neutral) | 25 days | (13, 52) | 24 days | (11, 49) | 24 days | (10, 48) |
| A | 21 days | (10, 44) | 19 days | (8, 40) | 18 days | (7, 38) |
| SA | 18 days | (8, 38) | 16 days | (6, 34) | 15 days | (5, 32) |

Q1, Q3 = first and third quartiles; Interpretation: A clear saturation gradient emerges by stance. Vaccine-hesitant videos (SA, A) saturate 44% faster than pro-vaccine videos (SIF) when measured by absolute differences (18 vs. 32 days for views). The gradient is consistent across all three engagement metrics, though the pattern is most pronounced for likes and comments. This temporal asymmetry implies a narrow window for effective public health intervention: while pro-vaccine content accumulates its audience over 4 weeks, vaccine-hesitant content achieves saturation within 2.5 weeks.

**H. Engagement Trajectories at Alternative Activity Thresholds**


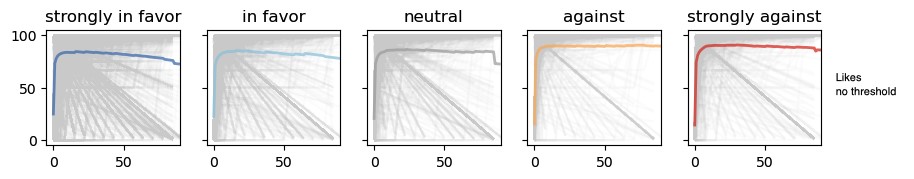

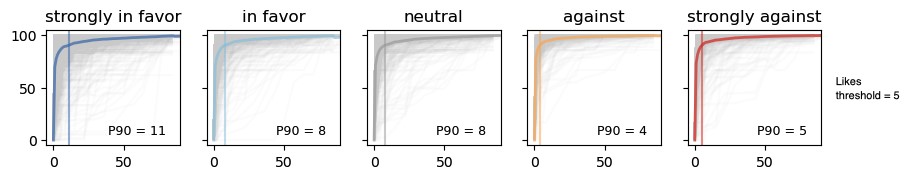

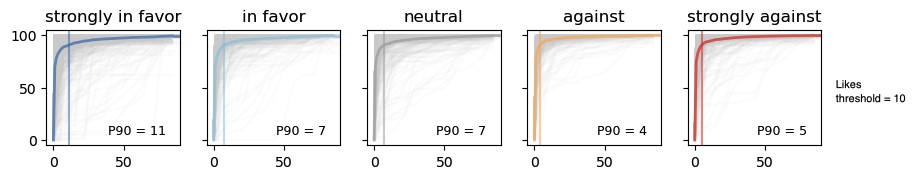

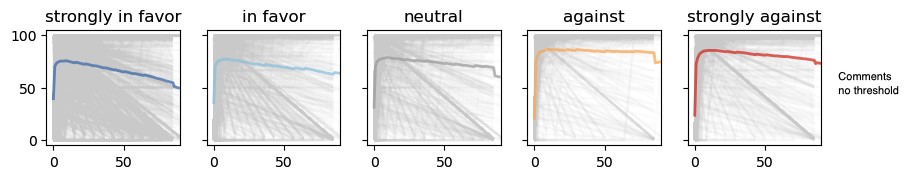

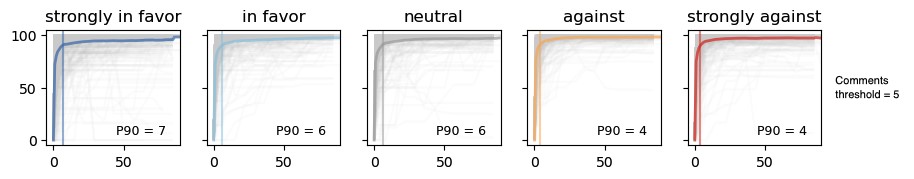

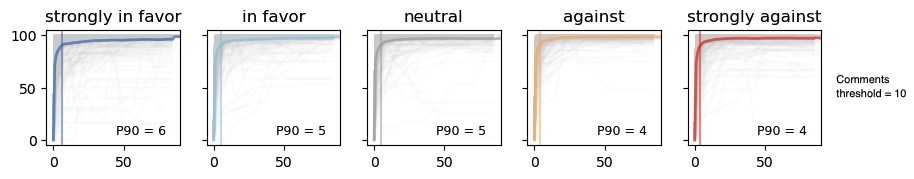


Figure S1: Like (top) and comment (bottom) trajectories, without engagement thresholding, and at thresholds of 5 and 10.

**I. Top 10 Channels**

Table S9. The number of videos in the dataset, channel name, distribution of video labels, and a description snippet for the top 10 channels contributing the most videos to the dataset. Labels are shown from strongly in favor (blue, left) to strongly against (red, right). The description snippets are from the channel's description at the time of the paper's writing. Below the channel name, our classification is included in square brackets (org - organization, ind - individual).

| **N_v_** | **Channel Name** | **Labels** | **Description Snippet** |
| --- | --- | --- | --- |
| 71 | HIV RNA Test Guide  [org, health] | 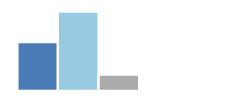 | HIVRNATestGuide is a platform where people can learn about common sexually transmitted diseases i.e STDs. [...] |
| 45 | CTV News  [org, news] | 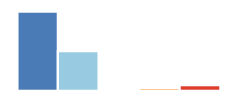 | CTV News is Canada's most-watched news organization both locally and nationally, and has a network of national, international, and local news operations. |
| 43 | Forbes Breaking News  [org, news] | 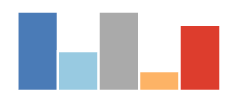 | [no description] |
| 40 | Dr. John Campbell  [ind, doctor] | 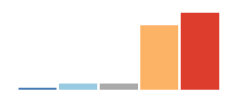 | My name is John Campbell and I am a retired Nurse Teacher and former clinical nurse based in England. I also do some teaching in Asia and Africa when time permits. [...] |
| 38 | Vejon Health [ind, doctor] | 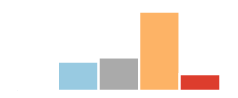 | At Vejon Health, we want to make the world a better place for you and future generations by improving the understanding and management of complex diseases. [...] |
| 29 | 9NEWS  [org, news] | 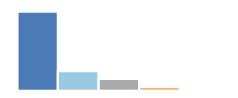 | Join 9News for the latest in news and events that affect you in your local city, as well as news from across Australia and the world. [...] |
| 29 | MicrobeTV  [ind, health] | 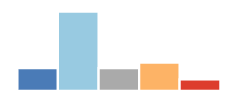 | I'm Vincent Racaniello, Earth's virology Professor, and I believe that education should be free. It's my goal to teach virology and other life sciences to the world. [...] |
| 26 | WFAA  [org, news] | 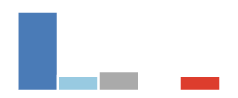 | The official YouTube channel of WFAA-TV and WFAA.com. |
| 26 | WION  [org, news] | 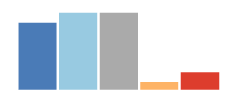 | WION -The World is One News, examines global issues with in-depth analysis. We provide much more than the news of the day. Our aim to empower people to explore their world. [...] |
| 25 | The Jimmy Dore Show  [ind, opinion] | 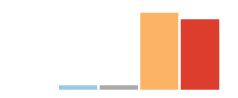 | Jimmy Dore is outrageous and outraged, bothersome and bothered. A crucial, profane, passionate voice for progressives and free-thinkers in 21st-century America. [...] |

**J. Engagement Statistics of Removed Videos**


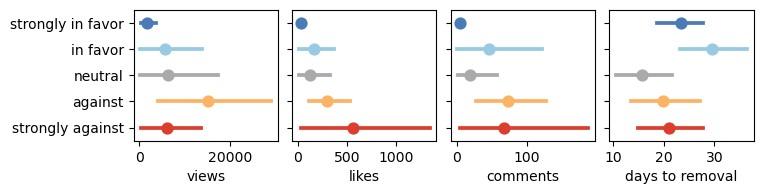


Figure S2: Distribution of views, likes, comments, and days to removal at the last collection period of videos that were removed.
